# Circulating Non-Coding RNAs as a Signature of Autism Spectrum Disorder Symptomatology

**DOI:** 10.3390/ijms22126549

**Published:** 2021-06-18

**Authors:** Salam Salloum-Asfar, Ahmed K. Elsayed, Saba F. Elhag, Sara A. Abdulla

**Affiliations:** Neurological Disorders Research Center, Qatar Biomedical Research Institute, Hamad Bin Khalifa University, Qatar Foundation, Doha P.O. Box 34110, Qatar; ssalloumasfar@hbku.edu.qa (S.S.-A.); aabdelaal@hbku.edu.qa (A.K.E.); sabaelmubarak@gmail.com (S.F.E.)

**Keywords:** ncRNAs, miRNAs, piRNAs, snoRNAs, Y-RNAs, tRNAs, lncRNAs, autism spectrum disorders, biomarkers, diagnosis, plasma

## Abstract

Autism spectrum disorder (ASD) is a multifaced neurodevelopmental disorder that becomes apparent during early childhood development. The complexity of ASD makes clinically diagnosing the condition difficult. Consequently, by identifying the biomarkers associated with ASD severity and combining them with clinical diagnosis, one may better factionalize within the spectrum and devise more targeted therapeutic strategies. Currently, there are no reliable biomarkers that can be used for precise ASD diagnosis. Consequently, our pilot experimental cohort was subdivided into three groups: healthy controls, individuals those that express severe symptoms of ASD, and individuals that exhibit mild symptoms of ASD. Using next-generation sequencing, we were able to identify several circulating non-coding RNAs (cir-ncRNAs) in plasma. To the best of our knowledge, this study is the first to show that miRNAs, piRNAs, snoRNAs, Y-RNAs, tRNAs, and lncRNAs are stably expressed in plasma. Our data identify cir-ncRNAs that are specific to ASD. Furthermore, several of the identified cir-ncRNAs were explicitly associated with either the severe or mild groups. Hence, our findings suggest that cir-ncRNAs have the potential to be utilized as objective diagnostic biomarkers and clinical targets.

## 1. Introduction

In the wake of the awareness and acceptance that autism spectrum disorder (ASD) is complicatedly heterogeneous and constantly rising [1], very little is known of its etiology and pathophysiology. According to the DSM-5 [2], current classifications of individuals with ASD house them under two core symptoms, which are: (1) deficits in social communication and social interaction and (2) restricted, repetitive patterns of behavior, interests, or activities. To date, subjective and clinical diagnosis has been the standard method of identifying children with the disorder, which has been helpful but is still far from ideal. This method risks late/missed diagnosis and, on occasion, ineffective therapeutic interventions [3]. Hence, finding alternative methods to objectively and systematically identify children with ASD, and assimilate a more compressive understanding of where an individual lies on the spectrum, is an important research area, paving the way to better therapeutic strategies and personalized medicine.

Biomarker discovery has increasingly been documented as an impactful avenue in disease diagnosis and is essential for drug discovery and restricting the advancement of diverse groups of diseases. The constant emergence of new and innovative technologies has made it even more possible to identify and validate biomarkers, making them a viable way to provide patients with personalized medicine. Furthermore, evidence has shown that the proper development and machinery of the central nervous system relies on the complex and finely orchestrated spatiotemporal expression of non-coding RNA (ncRNA), and their capricious regulation contributes to irregularities in gene regulator networks, brain development, and their correlating neurodevelopmental disorder [4]. Since ncRNAs impact biological processes, they could be utilized as attractive predictive and diagnostic biomarkers of ASD. Furthermore, due to the complexities and behavioral phenotypic heterogeneity of ASD, one must further understand the biological players that contribute to the disorder’s outcome and provide objective and reliable data to faction between these distinct groups and symptom severities. Ultimately, the goal is to cultivate tremendous leaps in diagnostic methods and manage personalized treatment strategies for ASD. This paper highlights the impact of utilizing cir-ncRNA biomarkers in ASD diagnosis and underlines an appreciation of their use as an emblem of ASD severity.

Mammalian cells have been recognized as investing energy in the production of endless diversities of small and large RNA transcripts that do not code for proteins, known as non-coding RNAs (ncRNAs). Traditionally, only ribosomal RNA (rRNA) and transfer RNA (tRNA) were considered ‘functional’ RNA, whereas all other non-coding transcripts were deemed ‘desert’ or ‘junk’ DNA. However, transcriptome data have expanded and shed light on the prominence and the seemingly infinite diversity and complexity of RNA biology, and their influence on numerous areas of cellular biology and molecular mechanisms that were once overlooked. Their ability to seemingly be secreted and circulated within exosomes, microvesicles, or RNA-binding proteins such as Argonaute 2, make them easily detectable and a less invasive biomarker of pathophysiological status [5]. Some examples of ncRNAs and their influential roles in gene regulation or interference include small nuclear RNAs (snoRNAs; RNA splicing), small nucleolar RNAs (RNA modification), miRNAs (RNA degradation and/or translation inhibition), PIWI-interacting RNAs (piRNAs; gene silencing), Y-RNAs (DNA replication), transfer RNAs (tRNAs), and long non-coding RNAs (gene regulation) [6]. Furthermore, ncRNAs were previously shown to be differentially regulated in neurodegenerative diseases [7], while others have suggested that its rich repertoire within the human brain makes their involvement and dysregulation potentially crucial in the steps that lead to abnormal brain development and the etiology of neurodevelopmental disorders [1,8].

miRNAs are small and single-stranded RNAs of approximately 22 nucleotides that have been identified as crucial post-transcriptional inhibitors of gene expression. miRNAs act as regulators in “fine-tuning” the translational output of target messenger RNAs (mRNAs) by promoting their degradation and inhibiting translation while regulating a plethora of physiological pathways and metabolic processes. The expression pattern of miRNAs is dynamically regulated during neurogenesis, neuronal differentiation, maintenance and function and overall brain development, making their involvement in neurodevelopmental processes of interest [1,9]. Studies have shown the critical role of alterations in miRNA regulation and signaling in many neurodevelopmental disorders, such as Fragile X syndrome, Rett Syndrome, Down Syndrome, and Prader-Willi Angelman Syndrome [8]. Since ASD has been associated with several of these conditions, it is interesting to investigate how miRNA dysregulation and/or expression patterns contribute.

Moreover, previous studies have proven that circulating miRNAs (cir-miRNAs) are emerging as significant biomarker for diagnosis and prognosis in human disorders and disease. It is understood that the miRNA repertoire that manifests itself in varying cell types is highly defined. In other words, the characteristic expression pattern of miRNA in different tissues, or cells, contributes to the shaping of specific features and functions. Some have even been identified as being solely expressed in particular tissues or cell types. Hence, this sheds light on the ability to identify specific miRNA expression patterns in varying diseases and disorders. Consequently, it has previously been shown that miRNA can be used as a diagnostic marker for certain types of cancers or cardiovascular conditions, as they contain specific profile characterizations [10].

Subsequently, this highlights the importance and potential of cir-miRNAs as viable and reliable biomarkers for ASD. Assessing the expression patterns of miRNAs and their influential capacity towards neurodevelopmental disorders is an exciting area of exploration. Previous investigations have underlined their effects on the spatial localization or compartmentalization of protein translation in dendrites, axons, and synapses [9]. Additionally, understanding the importance of their unique expression patterns can lead to new treatment strategies, in which miRNAs may be down-regulated, inhibited, up-regulated, or even replaced. In previous studies, multiple miRNAs have been identified as down- or up-regulated in blood, serum, and saliva, with the promise of their having relevance in ASD [11,12,13]. A meta-analysis review has recently shown the possibility of using oxytocin and vasopressin as biomarkers; however, this is heterogeneous in psychiatric disorders and does not provide convincing findings that peripheral OT or ADH levels are altered in ASD compared to healthy controls [14]. Moreover, a recent review yielded data on 67 environmental risk factors and 52 biomarkers. Only two associations, maternal overweight before or during pregnancy and SSRI use during pregnancy, might act as an independent risk factor for autism spectrum disorder [15].

Furthermore, another compelling group of non-coding RNAs are the piRNAs, which are characterized as having 24–31 nucleotides and have a role in regulating gene expression and stability, and silencing transposons by driving PIWI proteins of the Argonaute family towards specific genomic loci [8]. Once considered to be “dark matter”, piRNAs have emerged as potentially crucial regulators of disease outcomes, especially in cancer, and their aberrant expression is a distinct characteristic [16]. Furthermore, their value for use as a molecular marker has increased due to their abundance in various body fluids, their capacity to not easily be degraded by ribonucleases, and the fact that they are short enough to pass through cell membranes [7,17].

Additionally, snoRNAs are another fascinating group of non-coding RNAs which are characterized as metabolically stable RNAs that consist of 60–300 nucleotides and accumulate within the nucleoli. snoRNAs are subdivided into two main classes, which possess distinctive and evolutionary conserved sequence elements: C (RUGAUGA)/D (CUGA) motifs and H (ANANNA)/ACA elements, which guide the 2’-O-methylation and pseudouridylation of ribosomal RNAs (rRNAs), respectively, regulating its modification, maturation, and stabilization. Emerging evidence has further expanded our understanding of their roles, including endonucleolytic processing, guiding small nuclear RNAs and mRNA, and regulating alternative splicing in pre-mRNAs [18]. Previous investigations have shown that aberrant expression levels of snoRNAs, as well as mutations in its structure, influence physiological stress responses, including oxidative stress, tumorigenesis, metastasis, memory consolidation and learning, cholesterol, and another metabolic homeostasis, as well as being implicated in Prader–Willi syndrome, which has behavioral phenotypes that overlap with ASD [18,19]. Moreover, it is important to note that snoRNAs expression levels are stable and easily detectable in blood plasma, serum, and urine, making it a promising biomarker target.

To date, reliable biomarkers to diagnose ASD or define its severity do not exist. This study aims to comprehensively explore the potential of cir-ncRNAs as diagnostic biomarkers of ASD, as well as examine their expression profiles as a signature of ASD symptom severity. Our research plan used a sensitive, accurate, and cost-efficient methodology to profile ncRNAs. Consequently, we utilized next-generation sequencing (NGS) to identify novel non-coding species that could be functionally relevant as well as reliable biomarker targets. The dynamic expression pattern of cir-ncRNAs may be associated with the outcome of ASD and, further, distinguish between individuals that express either severe or mild symptoms of ASD.

## 2. Results

### 2.1. Cohort Characteristics and the Design of the Study

**Figure 1:**
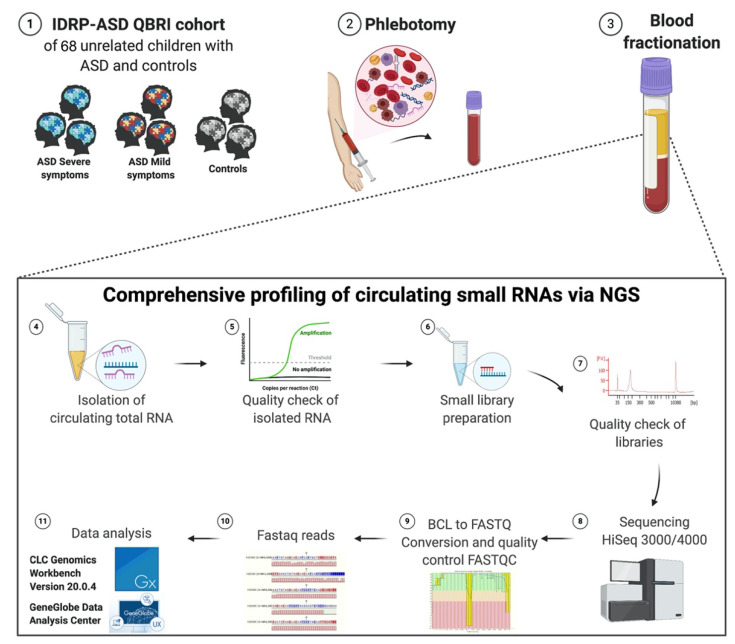
An overview of the experimental design of the study. (**1**) Sample selection; (**2**) Phlebotomy; (**3**) Blood fractionation; (**4**) RNA extraction and elimination of contaminants; (**5**) Assessment of isolated RNA and quality check using qRT-PCR; (**6**) Small library preparation; (**7**) Library quantification and assessment using bioanalyzer and Qubit; (**8**) Sequencing of libraries using NGS technology HiSeq 3000/4000 Illumina sequencing system. (**9**) BCL2 to Fastaq conversion and generation of Fastqc files. cDNA synthesis for small RNA without fragmentation; (**10**) Fastaq reads; (**11**) Data analysis using CLC Genomics Workbench program version 20.0.4 and Geneglobe Data analysis center. Created with BioRender.com.

Our pilot study analyzed a total of 60 children with ASD, and eight age- and gender-matched controls. All subjects included in this study were clinically assessed through a multidisciplinary team. Their assessment tools included the Diagnostic and Statistical Manual of Mental disorders (DSM-5), and the Autism Diagnostic Observation Schedule (ADOS). Accordingly, our 60 ASD subjects were divided into 35 children, who expressed more symptoms (severe) and 25 who expressed fewer symptoms (mild). The cohort characteristic description of ASD cases and controls are summarized in Table 1. Figure 1 illustrates the workflow followed in this study.

### 2.2. Sequencing the Circulating Transcriptome of ASD Cases that Expressed Mild and Severe Symptoms and Controls

Before initiating library preparation, we assessed the isolated RNA quality, as the Unique spike-ins and qPCR-based miRNA quality control are crucial for low-abundance RNA samples. The expression levels of fivr miRNAs (miR-103, miR-191, miR-30c, miR-451, and miR-23) and 3 out of the 52 added spike-ins were evaluated based on qRT-PCR Ct values (Table 2). As described in the methods section, calculating delta CT for UniSp100 and UniSp101 enabled us to distinguishing outlier samples. The delta CT for the two spike-ins ranged between 5 and 7. Furthermore, UniSp6 evaluates the cDNA synthesis, and its value should be <2 CTs between any two samples. Moreover, it is crucial to evaluate hemolysis in plasma biomarker identification studies; thus, the delta CT (miR-23a–miR-451a) was less than five, indicating that our RNA samples were of high quality. Endogenous control miRNAs in plasma (miR-103, miR-191, and miR-30c) were also detected in all samples.

Using the Qiaseq library preparation and sequencing protocol, we sequenced cell-free RNA, present in the plasma of ASD cases (with severe and mild symptoms) and controls. The library construction was optimized using different starting amounts of plasma for RNA extraction. We found that doubling the recommended starting amount of plasma (200 µL to 400 µL) improved the quality of the extracted RNA in the libraries.

### 2.3. Reliable Distribution and Abundance of Circulating Non-Coding Rnas within the Sample Cohort

**Figure 2:**
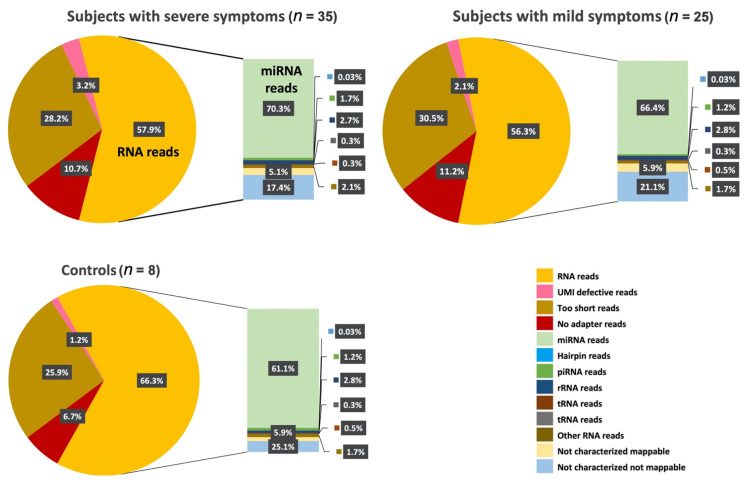
Circulating transcriptome profile analysis on plasma. Pie charts representing the relative abundance of RNA families in the plasma of controls (N = 8) and individuals that manifest severe symptoms of ASD (N = 35) and mild symptoms of ASD (N = 25). The group labeled as “Other RNA” in the pie charts is representative of reads derived from several Gencode annotation categories, such as snoRNAs, YRNAs, etc.

The QIAseq miRNA sequencing data were initially analyzed using the Qiagen GeneGlobe^®^ Data Analysis Center to ensure that the sequencing data had a reliable distribution. The reads were processed as follows: for each sample, 20–30 million reads were obtained, more than 55% of the reads were mapped to the human genome (hg19), and approximately 70% of these sequences were considered small RNA (sRNA), representing sequences between 18 and 43 nt. The pie charts (Figure 2) represent the relative abundance of RNA families in the plasma samples of the subjects that expressed more severe symptoms, subjects that expressed more mild symptoms, and controls. These results indicate that the RNAs that were extracted and identified are reliable, and the proportion or expression pattern of the RNAs within the target groups follows a similar distribution.

All reads assigned to a particular miRNA or piRNA ID were counted, and the associated UMIs were aggregated to count unique molecules. The largest category in terms of the frequency of reads was miRNAs, accounting for an average of 39.1% of reads (range 37.4–40.7%; Figure 2. The read counts and UMI counts were presented in the output Excel^®^ file “miR_piRNA” sheet. For sequences aligned with tRNAs or other RNAs, these results were displayed in the “tRNA” or “otherRNA” sheet, respectively. For the sequences aligned with the genome at the last alignment step (this is performed for humans using the most recent genome version), the same information (read counts and clustered UMIs) were output to the “notCharacterized_mappable” sheet. The remaining reads were also tallied (notCharacterized_notMappable) (Figure 2).

The Biomedical Genomics Analysis plugin in the CLC Genomics Workbench software was used to quantify the expression of each miRNA sample that was annotated and submitted to miRBase. Around 792 different human miRNA sequences were found in the samples, which accounted for approximately 1 × 10^6^ and 10 × 10^6^ reads for each sample.

The top 20 miRNAs, consisting of >70% of mapped miRNAs reads, were well-known, plasma-abundant miRNAs such as hsa-miR-16, hsa-miR-92a, has-miR-486-5p, hsa-miR-223, has-miR-122, members of the let-7 family (Table 3). The analysis was performed by the CLC Genomic Workbench software using the QIAseq miRNA differential expression analysis with slightly modified settings, which included a threshold to discard low background level intensities.

### 2.4. ncRNAs Differential Expression Analysis: ASD Cases vs. Healthy Controls

#### 2.4.1. Mirna Differential Expression Analysis: ASD Cases vs. Healthy Controls

**Figure 3:**
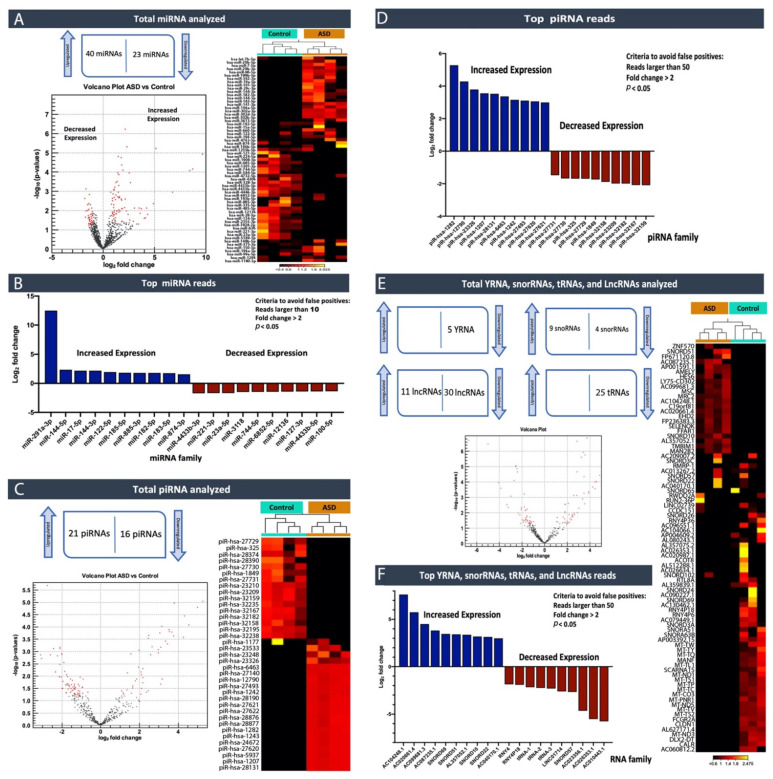
ncRNA expression analysis ASD individuals vs. Controls using CLC Genomics Workbench v20.0.4 (Qiagen, Germany)**.** (**A**) miRNA volcano plot (when using cutoff absolute fold change > 2, *p*-Value < 0.05, >10 reads per sample) and hierarchical clustering analysis of miRNA expression profile, a representative two-dimensional heatmap of expression values (4 Controls vs. 4ASD). Each column corresponds to one sample, and each row corresponds to a miRNA. The samples and features are both hierarchically clustered. (**B**) Top 10 differentially expressed miRNAs in ASD cases compared to control (Log_2_ fold change > 2; *p* < 0.05). (**C**) piRNA volcano plot (when using cutoff absolute fold change > 2, *p*-Value < 0.05, >50 reads per sample) and two-dimensional heat map of expression values. Each column corresponds to one sample, and each row corresponds to a piRNA. The samples and features are both hierarchically clustered. (**D**) Top 10 differentially expressed piRNAs in ASD cases compared to control. (**E**) Other ncRNA volcano plot (when using cutoff absolute fold change > 2, *p*-Value < 0.05, > 50 reads per sample) and two-dimensional heat map of expression values. Each column corresponds to one sample, and each row corresponds to a ncRNAs (Log_2_ fold change > 2; *p* < 0.05). (**F**) Top 10 differentially expressed other ncRNAs in ASD cases compared to control.

First, a miRNA differential expression analysis was performed between healthy controls and ASD groups. The analysis allowed for the identification of sixty-three miRNAs, differentially expressed between both groups, as shown in the volcano plot (when using cutoff absolute fold change > 2, *p*-value < 0.05, >10 reads per sample; Figure 3a). Forty miRNAs were identified as being differentially expressed between the groups with higher expression levels in ASD cases (fold change > 2; *p* < 0.05) (Table 4 and Supplementary Table S1). On the other hand, twenty-three miRNA showed significantly lower levels in the ASD cases compared to the healthy control (fold change < 2; *p* < 0.05) (Figure 3a, Table 4 and Supplementary Table S1). A two-dimensional heatmap of expression values showed a hierarchical clustering analysis of miRNA expressed in both groups; ASD vs. Controls (Figure 3A). The top ten up- and down-regulated miRNAs families with the largest change in expression are presented in the graph (Figure 3b). MiRNAs of the AAGUGCU seed family has-miR-291a-3p (hsa-miR-302b-3p, hsa-miR-302a-3p, hsa-miR-302d-3p) were to be highly significant upregulated in ASD cases.

#### 2.4.2. Other ncRNAs Differential Expression Analysis: ASD Cases vs. Healthy Controls

Initially, to assign reads to other small RNAs, such as piRNAs, the reads were mapped to piRNABank database Human_piRNA_sequence_v1.0 (accessed on 9 May 2020, regulatoryrna.org/database/piRNA/download.html). Among the 23,439 piRNAs species in the human genome, the differentially expressed piRNAs between the ASD cases vs. control groups were selected according to specific criteria. These criteria were as follows: the RPM (the number of reads per million clean tags) values are larger than 50, piRNAs should have at least a 2-fold difference in expression between the groups, and *p*-Value < 0.05.

A total of thirty-seven piRNAs were significantly changed in the ASD group, as shown in the volcano plot (Figure 3c). Furthermore, a clustering analysis of their expression profile indicates that twenty-one piRNAs were upregulated within the ASD group, while sixteen were downregulated. The highest upregulated piRNA was piR-has-1282 and the most significantly downregulated was piR-has-32159. Interestingly, there is a clear divide between the piRNAs expression between control and ASD groups, as shown in the heatmap (Figure 3c). This obvious demarcation makes piRNAs an up-and-coming area of exploration, which we are investigating further (Table 5; Figure 3c,d).

The unmapped reads from the QIAseq miRNA quantification workflow were collected and remapped to the whole human genome using RNA-seq analysis in CLC Genomics Workbench to assign reads to other non-coding RNAs, such as Y-RNAs, snoRNAs, tRNAs, and lncRNAs, that were present within the samples, showing significant changes in the expression profiles. Five YRNAs, four snoRNAs, thirty lncRNAs, and twenty-five tRNAs were downregulated. Meanwhile, nine snoRNAs and eleven lncRNAs were upregulated in ASD cases (Table 6; Figure 3e,f; Supplementary Table S2). These results also showed a clear divide between control and ASD cases. Figure 3f showed the top identified hits and clusters, which are under further investigation.

### 2.5. ncRNAs Differential Expression Analysis: Severe Symptoms vs. Mild Symptoms Cases

#### 2.5.1. miRNA Differential Expression Analysis: Severe Symptoms vs. Mild Symptoms Cases

**Figure 4:**
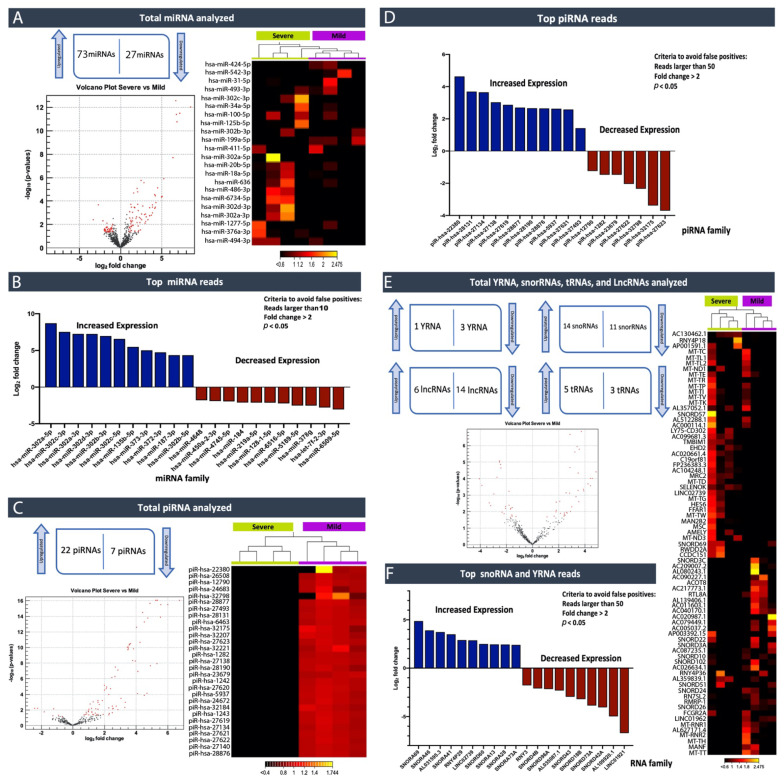
ncRNA expression analysis ASD individuals that manifest severe vs. mild symptoms using CLC Genomics Workbench v20.0.4 (Qiagen, Germany). (**A**) miRNA volcano plot (when using cutoff absolute fold change > 2, *p*-Value < 0.05, >10 reads per sample) and hierarchical clustering analysis of miRNA expression profile, a representative two-dimensional heatmap of expression values (4 Severe vs. 4 Mild). Each column corresponds to one sample, and each row corresponds to a miRNA. The samples and features are both hierarchically clustered. (**B**) Top 10 differentially expressed miRNAs in severe compared to mild (Log_2_ fold change > 2; *p* < 0.05). (**C**) piRNA volcano plot (when using cutoff absolute fold change > 2, *p*-Value < 0.05, >50 reads per sample) and two-dimensional heat map of expression values. Each column corresponds to one sample, and each row corresponds to a piRNA. The samples and features are both hierarchically clustered. (**D**) Top 10 differentially expressed piRNAs in in severe compared to mild (Log_2_ fold change > 2; *p* < 0.05). (**E**) Other ncRNA volcano plot (when using cutoff absolute fold change > 2, *p*-Value < 0.05, >50 reads per sample) and two-dimensional heat map of expression values. Each column corresponds to one sample, and each row corresponds to a ncRNAs. (**F**) Top 10 differentially expressed other ncRNAs in severe compared to mild.

The analysis identified one hundred miRNAs that were differentially expressed between the different symptomatology groups of ASD, as shown in the volcano plot (when using cutoff absolute fold change > 2, *p*-value < 0.05, >10 reads per sample) (Figure 4a; Supplementary Table S3). Seventy-three miRNAs were identified as being differentially expressed between the groups with higher expression levels in severe cases (fold change > 2; *p* < 0.05) (Table 7 and Supplementary Table S2). On the other hand, twenty-seven miRNAs exhibited significantly lower levels in the severe group compared to the mild group (fold change < 2; *p* < 0.05) (Table 7 and Supplementary Table S2). A two-dimensional heatmap of expression values showed a hierarchical clustering analysis of miRNA expressed in both groups with different symptomatology (Figure 4a). The top ten up- and down-regulated miRNAs families with the largest change in expression are presented in the graph (Figure 4b). Interestingly we observed that the miR-302 family (hsa-miR-302a-5p, hsa-miR-302c-3p, hsa-miR-302a-3p, hsa-miR-302d-3p, hsa-miR-302b-3p, hsa-miR-302c-5p and hsa-miR-302b-5p) were expressed at significantly high levels in individuals that expressed severe ASD symptoms in comparison to those that expressed mild symptoms. Previous findings have shown that the miR-302 family is crucial in stem cell pluripotency and renewal and somatic cell DNA demethylation [20,21,22].

Moreover, we noted that miR-135b-5p was expressed at high levels in ASD cases with severe symptoms vs. mild. Interestingly, it has previously been described that the variable regulation of disrupted in schizophrenia 1 (DISC1) by miR-135b-5p in the brain may prompt neuropsychiatric phenotypes [23].

#### 2.5.2. Other ncRNAs Differential Expression Analysis: Severe Symptom vs. Mild Symptom Cases

We analyzed whether piRNAs showed significant differences between severe and mild groups. As per our results, twenty-nine piRNAs were obtained based on the previously mentioned criteria, as shown in the hierarchical clustering analysis of the piRNA expression profile (Figure 4c,d; Table 8). Furthermore, the clustering analysis of the piRNA expression profiles showed that twenty-two piRNAs had upregulated expression levels in the severe group, while seven were downregulated piRNAs. The results indicate that piR-hsa-22380 is the most up-regulated piRNA (log2FC = 4.63) and piR-hsa-27623 is the most down-regulated piRNA (log2FC = −3.70) (Figure 4c,d; Table 8).

Additionally, we analyzed the Y-RNAs expression between both groups of different symptomatologies, and we succeeded in identifying four differentially expressed Y-RNAs; RNY3 (RNA, Ro60-Associated Y3), RNY3P1, RNY4 pseudogenes 28 (RNY4P28), and RNY4 pseudogenes 29 (RNY4P29), which were selected based on absolute fold-change >2 and *p*-value < 0.05 (Figure 4e,f; Table 9). The RNY4 pseudogene 29 (RNY4P29) expression level was significantly higher (logFC = 3.03) within the severe group compared to the mild group, whereas RNY3, RNY3P1, and RNY4P28 were noted to be significantly lower in the severe group.

Furthermore, according to our analysis, fourteen snoRNAs revealed greater expression in the plasma of individuals that expressed severe ASD symptoms, while eleven snoRNAs were downregulated. SNORA69 (also known as U69) was identified as the most up-regulated snoRNA (logFC = 4.63), and SNORD42A (U42) was identified as the most down-regulated (logFC = −3.70) (Figure 4e,f; Table 9). Additionally, we identified six upregulated lncRNAs and fourteen down-regulated lncRNAs, and, finally, five up-regulated tRNAs and three down-regulated tRNAs (Supplementary Table S4). These results also showed a clear divide between severe and mild ASD cases. The top hits and clusters identified are under further investigation. The potential to use piRNAs as a biomarker to faction between severe and mild symptoms is promising and may lead to personalized treatment strategies.

### 2.6. Pathway Enrichment by Ingenuity Pathway Analysis (IPA)—miRNAs

The dataset’s molecular networks, representing only the miRNAs with altered expression profiles, obtained from the CLC Genomic Workbench v20.0.4, were further analyzed via functional enrichment tests, using Ingenuity Pathway Analysis (IPA), for both ASD vs. controls and ASD with severe symptoms vs. mild analyses.

#### 2.6.1. Pathway Analysis in ASD vs. Controls Groups

Analysis of the significant miRNA pathways analysis that were identified mainly linked them to diseases and disorders associated to psychological and neurological disorders, and their molecular and cellular functions, cell growth, and movement (Table 10).

The network analysis in the IPA system searched for pathway crosstalk analysis and significant molecular networks. A total of three significant molecular networks were identified by Fisher’s exact test in the IPA system, with additional criteria specifying that a pathway’s score was at least 20 and each pathway had at least 10 molecules (Supplementary Table) observed sixteen ASD miRNAs candidates, enriched by the functions of neurological and psychological disorders. We highlighted Dickkopf WNT Signaling Pathway Inhibitor 1 (**DKK1**), EPH Receptor B6 (**EPHB6**), Neurotrophic Receptor Tyrosine Kinase 3 (**NRTK3**), and Tumor Protein P53 (**TP53**), which were previously described as being regulated by these miRNAs and involved in either brain disorders or ASD [24,25,26,27].

In addition to the significant network, other crosstalk networks and predicted molecules are noteworthy (Supplementary Table S3), such as **AGO2**, **BRAF**, and **NR4A2**. **AGO2** (Argonaute 2); **AGO2** mutations in some individuals have shown disturbances in neurological development and ASD [28,29]. Furthermore, B-Raf Proto-Oncogene, Serine/Threonine kinase (**BRAF**) is essential in synaptic transmission and plasticity, neuronal function, and the development of learning/memory [30,31,32]. Finally, Nuclear Receptor Subfamily 4 Group A Member 2 (**NR4A2**) is associated with intellectual disability and ASD [33].

#### 2.6.2. Pathway Analysis in ASD with Severe Symptoms vs. Mild

**Figure 5:**
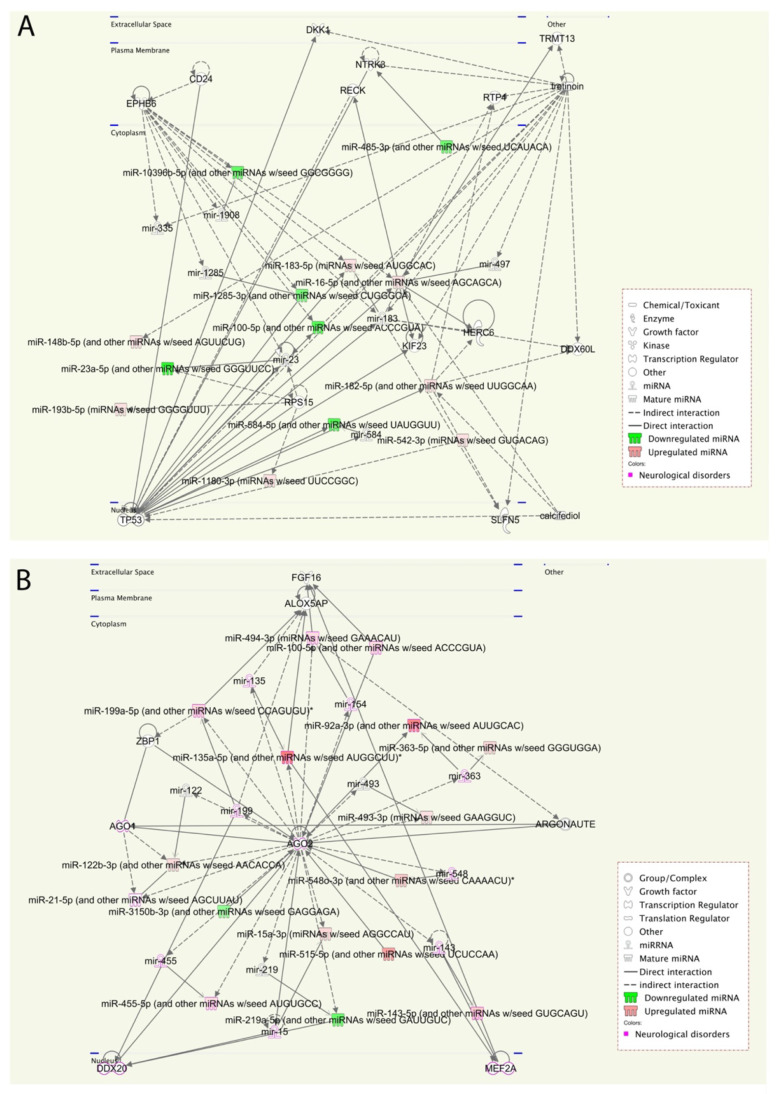
Most highly rated network through IPA analysis in (**A**) ASD cases vs. Controls and (**B**) ASD cases with severe symptoms vs. mild. (**A**) The network representation of the most highly rated networks [Neurological Disease, Organismal Injury and Abnormalities, Psychological Disorders] and (**B**) [Gene Expression, Organismal Injury and Abnormalities, Reproductive System Disease]. The genes that are shaded were determined to be significant from the statistical analysis. The genes shaded red are upregulated and those that are green are downregulated. The molecules highlighted in pink are those identified in neurological disorders. The intensity of the shading shows to what degree each gene was up- or down-regulated. A solid line represents a direct interaction between the two gene products and a dotted line represents an indirect interaction.

As the same findings were found in the comparison between ASD and control groups (Figure 5A), the significant pathways analysis showed that the miRNAs are essential and enriched in the psychological and neurological disorders, and cell regulation and development (Table 10). Here, a total of three significant molecular networks were identified by Fisher’s exact test in the IPA system, with additional criteria specifying that a pathway’s score was at least 20 and each pathway had at least 10 molecules (Supplementary Table S5). Figure 5B illustrates the significant grids related to the Gene Expression, Organismal Injury and Abnormalities, and Reproductive System Disease Network. In this network, we observed forty ASD miRNAs candidates, enriched with the functions of neurological and psychological disorders (Supplementary Table S6).

We highlighted **AGO2**, previously identified in the pathway analysis ASD vs. Control, which seems specific to individuals with severe symptoms of ASD. Additionally, Myocyte Enhancer Factor 2A (**MEF2A**) regulates cellular morphology and neuronal connectivity and mitochondrial functioning in neurons [34].

In addition to the significant network, there are other crosstalk networks and predicted molecules that are noteworthy, such as phosphatase and tensin homolog protein (**PTEN**) and B-Raf Proto-Oncogene, Serine/Threonine kinase (**BRAF**), which are essential in synaptic transmission and plasticity, neuronal function, and the development of learning/memory [30,31,32]. This finding complements prior knowledge of ASD phenotypes, providing further evidence of this disorder’s neuro-related processes (Supplementary Table S3). Moreover, Epidermal Growth Factor Receptor (**EGFR**) is associated with symptom severity in children with ASD [35]. Additionally, Insulin-Like Growth Factor 1 (**IGF-1**) is a neurotrophic polypeptide crucial in central nervous system growth, development, and maturation. Moreover, IGF-1 has emerged as a potential target for therapeutic approaches in several neurodevelopmental disorders, including ASD [36].

Furthermore, previous findings have shown that, in children with ASD, stimulation with **TLR2** has led to a high pro-inflammatory response [37]. ASD pathogenesis and symptom severity are thought to arise from complex interactions, including immune-inflammatory pathways and mitochondrial dysfunctions [38].

## 3. Discussion

It is imperative to appreciate that the accurate and early diagnosis of neurodevelopmental disorders such as ASD, and timely intervention, can potentially alter the adverse developmental trajectories and characteristic severities. At present, a reliable biomarker or a combination of biomarkers that can be used for precise ASD diagnosis does not exist. Consequently, ASD is mainly identified through behavioral phenotypes and characteristics. This subjective analysis leaves room for misdiagnosis and potentially ineffective treatment strategies. Therefore, there is an essential need to uncover specific and reliable biomarkers that provide objective identification of ASD and better faction the spectrum to deliver more effective therapies. The recruitment of very young, healthy children and children with ASD to donate blood proved to be difficult. Consequently, the small number of controls recruited is a limitation of our study. However, our analysis shows significantly associated circulating non-coding RNAs (cir-ncRNAs) specific to individuals with ASD, and their roles were reinforced through the pathway analysis results, despite the small sample size of controls.

The non-coding genome is significantly larger than the genome’s protein-coding portion; more than 98% of the human genome does not encode proteins [39]. Non-coding RNAs (ncRNAs) have a high potential to circulate in the blood and act as novel biomarkers for several conditions. Furthermore, cir-ncRNAs have recently been categorized as potential diagnostic markers for different conditions, including neurological disorders. Although there have been multiple studies associating circulating miRNAs with ASD, other non-coding RNAs have not been studied, and disorder causal genes have yet to be confirmed [1]. Additionally, several studies have examined miRNAs in whole blood [12], serum [13], and saliva [11], obtained from children with ASD compared to controls, but none of these studies isolated RNAs from plasma.

**Figure 6:**
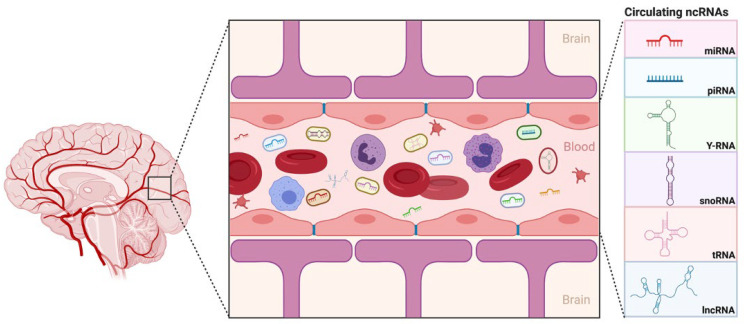
Executive Summary. Circulating ncRNAs as biomarkers, isolated from plasma, that can be used to distinguish severe ASD cases from mild ones and hence could be possibly applied as early diagnostic biomarkers. Created with BioRender.com.

Recent findings have indicated the biological variable that impacted miRNA profiles in plasma and serum. These two biofluids may exhibit some differences in their miRNA contents, notably due to the coagulation process occurring during serum collection [40]. The extent of platelet depletion in plasma samples is a critical variable, as well as the loss of cir-ncRNA through surface adsorption on the blood clot or through additional RNA degradation paired with the release of platelet-containing RNAs upon coagulation. In our study, we used platelet-depleted plasma, generated immediately after blood collection, which constitutes the preferred sample-collection process for cell-free circulating RNA biomarker discovery [41]. To the best of our knowledge, our study is the first to use plasma obtained from children with ASD to delineate ncRNA as a diagnostic biomarker. We have utilized comprehensive, evidence-based data sources to introduce, for the first time, several potential circulating ncRNAs biomarkers, isolated from the plasma, with the aim of identifying ASD cases that tend to manifest severe symptoms of ASD vs. mild ones, and hence, this may even be implemented as a tool in early diagnosis and treatment approaches (Figure 6).

In this study, by profiling ncRNAs in the plasma of ASD individuals, we examined several key ncRNAs biotypes; miRNAs (the most abundant biotype), piRNAs, snoRNAs, Y-RNAs, tRNAs, and lncRNAs. Each group of subjects (ASD vs. Controls and ASD individuals that expressed severe symptoms vs. mild symptoms) appeared to have differences in terms of the circulating ncRNAs expression profiles. In particular, within the miRNA family miR-302, which displayed substantially high read counts, we observed that hsa-miR-302a-5p, hsa-miR-302c-3p, hsa-miR-302a-3p, hsa-miR-302d-3p, hsa-miR-302b-3p, hsa-miR-302c-5p and hsa-miR-302b-5p were expressed at significantly high levels in cases where individuals exhibited severe symptoms of ASD compared to those that expressed a few or milder forms of their defining characteristics. This finding, miR-302a in plasma, has confirmed Vasu et al.’s (2014) findings that miR-302a is differentially expressed in the serum of ASD. Moreover, miR-302a seems to be highly expressed in ASD cases with severe symptoms. The significant increase in the miR-302 family sparked our interest, as miR-302 was previously shown to negatively influence the neural development, neuronal receptors, and synaptic plasticity [42]. We, therefore, aim to understand why children with higher expression levels of this family of miRNA show more severe symptoms. Consequently, miR-302a might be a biologically and analytically attractive biomarker for ASD with a severe symptomatic biomarker. Moreover, consistent findings have shown that the miR-302 family is critical in stem cell pluripotency and renewal, and somatic cell DNA demethylation [20,21,22]. miR-135b-5p was another miRNA that was flagged in our study as being expressed at significantly higher levels in individuals who expressed severe symptoms versus mild ones. It was previously described that variable regulation of disrupted in schizophrenia 1 (DISC1) by miR-135b-5p in the brain may predispose one to neuropsychiatric phenotypes [23]. Furthermore, a recent study has shown that miR-135 can serve as a biomarker of post-traumatic stress disorder (PTSD) and might be an important therapeutic target for dampening persistent and stress-enhanced memory [43]. By analyzing these miRNA pathways further, and identifying proteins the associated with them, one can then develop clinical assays that can be used to diagnose objectively.

Furthermore, we believe that simultaneous measurements of RNA and proteins can increase detection specificity for ASD and revolutionize the current diagnosis paradigm. We aim to develop an assay which simultaneously interrogates large sets of RNAs and proteins, as a unique, sole biomarker for ASD is near impossible.

We performed a pathway enrichment analysis to better understand the miRNA’s biological implications in the context of the regulatory system. The pathway analysis revealed that a large number of pathways are neuro-related processes, and several enriched molecules/proteins are involved, such as DKK1, EPHB6, NTRK3, P53, AGO2, PTEN, and BRAF. DKK1 has been previously described as being involved in the brain [25], whereas EPHB6, which plays important role in gut homeostasis, is generally considered as a candidate gene for ASD [26]. Furthermore, a genetic association was found between the NTRK3 gene and both autism and Asperger syndrome [27]. Additionally, altered P53 and p53-dependent pathways have been reported in autism [24]. AGO2 mutations in some patients contribute to disturbances in terms of neurological development and the outcome of ASD [28,29]. In our cohort, AGO2 was identified in the pathway analysis of ASD vs. Control and was found to be more specific to individuals that expressed more severe symptoms of ASD. Consequently, we will explore the role of AGO2 further. These results are consistent with prior knowledge of ASD phenotypes, providing new insights into interpretations of the underlying molecular mechanisms in ASD.

The relevance of ncRNAs, especially miRNAs, to human diseases and disorders was initially extensively studied in cancer [16,17,18,44,45]. Besides miRNAs, other ncRNAs, such as PIWI-interacting RNAs (piRNAs), act as key elements in cellular homeostasis and are crucial in transposon silencing during embryo development [46]. While cir-miRNAs were proven to be highly stable in the blood, piRNAs have also emerged as being stably expressed in circulation [40]. Remarkably, specific piRNAs have been useful in distinguishing between tumors and non-tumor tissues [16,17], and have been suggested to contribute to colorectal cancer development and risk, a concept of identification and exploration that may transcend into potentially aiding in a more objective classification of individuals within the ASD spectrum. Consequently, this suggests that it is an emerging, highly promising diagnostic and prognostic biomarker discovery and intervention area. Our study identified piRNAs that could, hypothetically, be used to help clinicians better faction between individuals, furthering more personalized treatment approaches.

Excitingly, a growing number of isolation methods for profiling cir-ncRNAs are being developed, making this a captivating and promising area of research. One of the significant advantages of deep sequencing platforms is its ability to identify a considerable number of ncRNA transcripts. As we have shown in our study, high-throughput sequencing revealed the existence of other classes of ncRNAs, including snoRNAs, Y-RNAs, tRNAs, and lncRNAs, indicating a wide range of regulatory RNAs with a wide variety of processing mechanisms and functions.

Using small RNA high-throughput sequencing, we demonstrated that the ~110 nucleotides (nt) long Ro-associated Y-RNAs (also called RNYs or Y-RNAs) are present in plasma. Our investigations elucidated that Y-RNA, hY3, and pseudogene hY3P1 are differentially downregulated in individuals with more severe symptoms of ASD. RNY4 Pseudogene 28 and 29, were further identified to be differentially expressed in the severe symptom group. Y-RNAs have emerged as being important for the initiation of chromosomal DNA replication, RNA stability, and cellular responses to stress [47]. As seen in the other types of non-coding RNA, past investigations into Y-RNA have mainly focused on cancer research, where an accumulation of investigations have shown that fragments of Y-RNAs displayed significant differential expression patterns, in both circulation and/or in tumor tissues as compared to controls [48]. This concept is exciting, as this holds promise regarding the role of Y-RNA as a biomarker in ASD, providing a platform to differentiate between ASD’s characteristic and symptom states, as well as, potentially, other neurodevelopmental disorders that may share similar symptoms to ASD. However, Y-RNA research is relatively new, and more in-depth research is required to better understand its functioning.

On the other hand, snoRNAs also offer novel insights into, and promising perspectives for, ASD. According to our analysis, the SNORA69 (known as U69) is the most up-regulated small nucleolar RNA, whereas SNORD42A (U42) is the most down-regulated snoRNA in individuals that expressed more severe ASD symptoms. Interestingly, a microdeletion of a subtype of snoRNA (HBI-85), has been previously associated with Prader–Willi-Like Syndrome (PWLS) [8]. Since PWLS has overlapping ASD characteristics (i.e., social difficulties), it is exciting to note the potential to utilize non-coding biomarkers as an objective identifier between similar neurodevelopmental conditions, further bringing us closer to better treatment strategies. While the exact roles of SNORA69 and SNORD42A were not specifically unveiled in the field of ASD, this suggests a promising area of investigation.

Collectively, our study supports and builds on other contributions in the field. To our knowledge, our study is the first to evaluate cir-ncRNAs in the context of two groups of symptom severities, in order to objectively identify differentiating flags between groups. Our study and consequent findings aim to improve on the currently existing platform of diagnosing children with ASD. Due to the complexities associated with ASD, one must develop a system to further appreciate the biological players that contribute to its molecular and phenotypic outcome. Furthermore, one must stress the importance of identifying objective and reliable data to faction ASD severities and characteristics within the spectrum and not solely rely on diagnostic comparisons of cases vs. controls. Opening our eyes to the possibility of assessing ASD within its spectrum is as essential as assessing ASD as compared to other neurodevelopmental conditions or control groups. This would potentially lead to even greater and exceptional management of personalized treatment strategies and outcomes.

Our capacity to truly understand ASD is limited by the subjectivity of analysis. While it is unlikely that one specific ncRNA is a factor between an individual without ASD and one with ASD, identifying cir-ncRNAs as antecedent biomarkers to identify the risk of developing ASD, as well as utilizing them as a tool to screen those already diagnosed with ASD and objectively assess ASD severity could revolutionize the current paradigm. In general, our pilot study identified ncRNAs as biomarkers indicative of altered neurodevelopment, and this impairment was measurable via alternations in their levels. Moreover, while we have identified specific ncRNAs that have significant expression levels between ASD and control, or within severe vs. mild symptomology groups, we believe that a panel of biomarkers, whether they are within an identified non-coding family, in combination with other ncRNA, or in association with other proteins, would provide a higher degree of specificity and sensitivity in terms of diagnosis, compared to a single biomarker.

Finally, while this study did not characterize the biological functions of cir-ncRNAs in vitro, it did substantiate a very sensitive methodology for profiling all circulating extracellular RNAs and explored their functional relationships using sophisticated, pathway-based analysis tools. The study suggests that one should prioritize the identified candidate ncRNAs for further validation in a larger cohort and perform rigorous functional studies.

## 4. Materials and Methods

### 4.1. Subjects—The Interdisciplinary Research Program (IDRP) ASD Cohort

The samples utilized in this study were obtained from a depository belonging to Qatar Biomedical Research Institute (QBRI) Interdisciplinary Research Program (IDRP) entitled Identifying Potential Molecular Biomarkers for ASD. The umbrella study encompasses various disciplines and a blend of omic investigations to further our understanding of the fundamental underpinnings of ASD and establish diagnostic tools for its early detection. Children ranging in age from 3 to 15 years were recruited, alongside their parents. Recruitment was solely based on families that reside in Qatar. ASD cases were subdivided based on characteristic symptoms of ASD or were diagnosed as having ASD with an associated comorbidity (i.e., attention-deficit/hyperactivity disorder (ADHD), intellectual disability (ID), or epilepsy). This study’s strength lies in the varying attributes used to define the divisions within the cohort based on the absence of communication and social skills, symptomatology, and comorbidities. Age-matched control groups included siblings/healthy individuals from the general population and a neurodevelopmental disorder group of age-matched children that solely elicited ADHD, ID, or epilepsy.

For our current pilot study, we subdivided children into those that exhibited severe (*n* = 35), mild symptoms (*n* = 25), and controls (*n* = 8). The clinical characteristics of the subjects are described in Table 1.

### 4.2. ASD Assessment

Children were clinically assessed and diagnosed with ASD at the Rumailah Hospital and Shaffalah Center for Children with Special Needs, Doha, Qatar. All children were diagnosed through a specialized, multidisciplinary team (MDT) consisting of medical doctors, psychiatrists, clinical nurse specialists, community mental health nurses, psychologists, social workers, and occupational therapists. Furthermore, validated screening and diagnostic tests and tools, including the Diagnostic and Statistical Manual of Mental disorders (DSM-5), Autism Diagnostic Observation Schedule, Second Edition (ADOS), and Autism Diagnostic Interview-Revised (ADI-R), were used.

### 4.3. Severity Classification

To ensure that the analyzed samples were grouped accordingly, the following criteria was used: (1) ADOS-2 score to verify the initial clinical diagnosis, (2) DSM-5 severity levels, and (3) clinical assessment by experienced and qualified clinicians. Due to the complexity and heterogeneity of ASD, classifying an individual as having the disorder is a perplexing endeavor. Hence, to in order respect and be sensitive to the extensive and multifaced classification of ASD diagnosis, we have divided our findings into two groups, the first of which represents individuals that exhibit severe symptoms, displaying multiple, unambiguous characteristics of ASD, including severe behavioral phenotypes (i.e., significant alternations in social and language development), and those that show mild symptoms of ASD.

### 4.4. Collection of Human Blood/Plasma

The collection of blood samples complied with the national guidelines that oversee research investigations comprising vulnerable subjects such as children. Well-trained phlebotomists, with extensive experience working with children with special needs, were responsible for collecting venous blood samples. Furthermore, an EMLA cream was used with the local anesthesia to avoid and/or reduce pain sensitivity during blood withdrawal. Samples were collected into VACUETTE^®^ tubes containing EDTA, centrifuged at 1800 rpm for 10 min, followed by plasma collection and re-centrifugation for 10 min at 3000 rpm. Finally, plasma samples were aliquoted into 200 μL aliquots and stored at −80 °C until further use.

### 4.5. RNA Isolation from Peripheral Blood Plasma

Frozen plasma samples were thawed in a 37 °C water bath. The thawed plasma samples were centrifuged at 400× *g* (~2000 rpm) for 2 min to remove cells and precipitated plasma proteins/lipids. According to the manufacturer’s instructions, cell-free (cf) plasma samples were transferred to new tubes for RNA isolation using the miRNeasy Serum/Plasma Advanced Kit (Cat. no. 217204, Qiagen, Germany). We optimized the recommended starting amount of plasma; due to the low quantity of cfRNA, we used 200 μL of plasma for total RNA extraction, with the addition of 52 Qiaseq miRNA Library QC Spike-ins (Cat. no.: 331541, Qiagen, Germany) as an internal control for miRNA expression profiling in plasma.

### 4.6. QIAseq miRNA Library Quality Check

The QIAseq miRNA Library QC qPCR Assay Kit (Cat. no. 331551, Qiagen, Germany) was used to evaluate RNA isolation quality before small RNA library preparation and to assess NGS performance post-sequencing. The kit provides 52 Spike-Ins controls with a qPCR panel that monitors the technical quality of the whole process from RNA isolation (by evaluating the reproducibility) to sequencing data analysis (by checking the reads). This method also enables the detection of enzymatic inhibitors or nucleases and hemolysis assessment (necessary for plasma miRNA identification). In brief, the procedure started during RNA isolation with the addition of 52 QIAseq miRNA Library QC Spike-Ins to the samples. The sample evaluation was determined using qRT-PCR. For the identification of RNA isolation efficiency, the calculation of delta CT for UniSp100 (CT: 31–34 range) and UniSp101 (CT: 25–28 range) was assessed and found to be within the recommended value of around 5–7. For the inhibitor detection, the UniSp6 was measured. The value was <2 CTs between any two samples. For hemolysis, delta CT (miR-23a–miR-451a) was less than 5, ensuring high-quality samples. A value of 5–7 was considered a borderline sample. Samples with a value > 7 were not used.

### 4.7. Small RNA Library Preparation

For the library construction and molecular indexing, the QIAseq miRNA Library Kit (96) (Cat. no. 331505, Qiagen, Germany) and QIAseq miRNA NGS 96 Index IL (Cat. no. 331565, Qiagen, Germany) were used. The gold standard approach for the normalization of circulating miRNAs utilizes equal amounts of biofluids and isolated total RNA, and the spike-ins are used as normalization controls. Thus, 5 μL of 15 μL total RNA column eluate was used for library preparation. RNA samples were subjected to 3′ and 5′ adaptor ligation targeting miRNAs by reverse transcription to generate the cDNA construct based on small RNA having 3′ and 5′ adaptor ligation. This reverse transcription step will help to enrich the RNA fragments with 3′ and 5′ adaptors on both ends. The reverse transcription (RT) primer contained an integrated Unique Molecular Indices (UMI). The RT primer binds to a region of the 3′ adapter and facilitates conversion of the 3′/5′ ligated miRNAs into cDNA while assigning a UMI to every miRNA molecule. During reverse transcription, a universal sequence is also added. The sample indexing primers recognize this during library amplification. cDNA constructs were purified using a streamlined magnetic bead-based method. Then, the unbiased amplification of libraries was accomplished using a dried universal forward primer from a plate paired with 1 of 96 dried reverse primers in the same plate (Cat. no. 331565, Qiagen, Germany).

Consequently, this assigned each sample a unique custom index. After the library amplification, a cleanup was performed, using the streamlined magnetic bead-based method. Validation of the libraries was performed using Agilent technologies 2100 Bioanalyzer with an Agilent High Sensitivity DNA assay (Cat. no. G2938-90020, Agilent Technologies, United States). A unique peak of around 141 bp was obtained (a purified library example is shown in Figure 1).

### 4.8. Small RNA Deep Sequencing

cDNA libraries were measured based on the average size obtained from the bioanalyzer using the Qubit Fluorometer, Qubit HS dsDNA Assay Kit (Cat. no. Q32854, Thermo Fisher Scientific, United States). Libraries were diluted to 10 nM using a resuspension buffer and pooled with unique indexing for Illumina. The final dilution loaded was 3nM, with further clustering on cBot2, and sequencing on the Illumina platform was achieved using the HiSeq 3000/4000 SBS Kit (150 cycles). To discover novel miRNAs, we aimed to generate up to 20 million reads per sample. The adapters were trimmed. The raw data from the Illumina HiSeq 3000/4000 were converted from bcl2 to fastq format.

### 4.9. Sequencing Read Mapping and Small RNA Annotation

The raw sequence files from the Illumina HiSeq 3000/4000 in the *BCL* format were converted to the *FASTQ* format using the bcl2fastq v1.8.4 conversion tool software (Illumina, United States). Reads were filtered and adapters were trimmed. After adapter trimming, the read data were evaluated for quality using FASTQC to filter out reads with a quality score [49].

### 4.10. UMI Analysis: The Geneglobe Data Analysis Center

The GeneGlobe data analysis center (accessed on 18 February 2020, www.qiagen.com/us/shop/genes-and-pathways/data-analysis-center-overview-page/) can align and report on the QIAseq miRNA spike-ins in addition to the aligned small/miRNA/piRNA from each sample. This QIAGEN analysis tool was used to assess the effectiveness of QIAseq’s UMIs. For the synthetic miRNA samples, the option ‘other’ was chosen for mapping, while ‘human’ was chosen for the human total RNA samples during the primary data analysis. The resulting count table included UMI and raw read counts for each miRNA in the samples. Before analyzing the correlation between UMI and raw read counts, the counts were rlog transformed.

Next-generation sequencing (NGS) allows not only the quantification of known miRNAs, but also the identification and quantification of novel miRNAs, isomiRs (miRNA variants), and other small RNA species that can be functionally relevant in diseases and is therefore used as potential disease biomarker (Figure 2). miRNAs are identified by aligning the reads to miRBase (version 21), and the reads are tallied to generate total counts for each miRNA. Statistical significance (*p*-Value) between two or more samples was calculated to generate differential expression profiles.

### 4.11. Differential Expression Analysis: CLC Genomics Workbench Version 20.0.4

Files were then exported to the CLC Genomics Workbench (version 20.0.4) for read mapping to the hg38 human genome version. This allowed for a single-mismatched base, down to 18 nucleotides. Analysis of the resulting data was performed using small RNA analysis tools in CLC Genomics Workbench. Spike-in reads were filtered out from the rest of the data. A “perfect match” setting was applied when mapping, filtering, and counting QIAaseq NGS Spike-in reads in a dataset. After counting the QIAseq NGS Spike-in reads, they should be normalized to the total number of reads per sample. After this normalization, correlation matrices should be plotted for all sample-to-sample comparisons. This is done to evaluate the sample-to-sample correlation in the sample set. The expected correlation should be R2 of 0.95–0.99. If samples deviate from these values, they could be technical outliers, and potentially excluded from downstream analysis.

Using the Biomedical Genomics Analysis plugin that supports the analysis of reads sequenced using the QIAseq miRNA Library Kit, the QIAGEN miRNA Quantification workflow quantified the expression in each sample miRNAs found in miRBase. Reads were first mapped to miRBase version 21 database (accessed on: 9 May 2020, www.mirbase.org) and piRNABank database Human_piRNA_sequence_v1.0 (accessed on: 9 May 2020, www.regulatoryrna.org/database/piRNA/) to assign reads to miRNAs and piRNAs, respectively, and to exclude them before mapping to the whole human genome. The unmapped reads from the QIAseq miRNA quantification workflow were collected and mapped using RNA-seq analysis to assign reads to other non-coding RNAs, such as Y-RNAs, snoRNAs, tRNAs, and lncRNAs.

The QIAseq miRNA Quantification tool allows for the grouping of miRNA into mature miRNA; the same mature miRNA may be produced from different precursor miRNAs, or on seed, as the same seed sequence may be found in different mature miRNAs. A custom database for piRNAs was also provided. This miRNA grouped on seed was used for further analysis through the Ingenuity Pathway Analysis (IPA) platform. The workflow calculates differential expressions for expression tables with associated metadata using multi-factorial statistics based on a negative binomial Generalized Linear Model (GLM). Both Grouped on Mature and Grouped on Seed expression tables can be used. Integrated Unique Molecular Indices enable the quantification of individual miRNA molecules, eliminating PCR and sequencing bias. For the differential expression analysis, miRNAs were deemed to be statistically differentially expressed if they had an expression of greater than 50 read counts at an absolute fold change > two and an adjusted *p* < 0.05.

### 4.12. Functional Enrichment Tests

We used the Ingenuity Pathway Analysis (IPA) system for pathway analysis and molecular networks to perform the candidate miRNAs’ functional enrichment tests. The IPA system provides a more comprehensive pathway resource based on manual collection. The rich information returned by IPA is also suitable for pathway crosstalk analysis, as it has almost all molecules, including their connections. Briefly, the IPA system implements Fisher’s exact test to determine the pathways that are enriched with miRNAs of interest. Furthermore, the IPA system’s network analysis searches for significant molecular networks in a commercial knowledge base, including integrative information from the literature, gene expression, and gene annotation.

## 5. Conclusions

It is crucial to bear in mind that the early detection of neurodevelopmental disorders such as ASD, and its timely intervention, could potentially alter the adverse developmental trajectories and severities associated with them. Our current understanding of ASD still requires extensive exploration. Unraveling the fundamental underpinnings of the disorder will meritoriously lead to more effective treatment interventions. To date, current aid focuses mainly on improving behavior, and while this has been proven to help somewhat, it does not address the core of the disorder. Furthermore, this crutch has created a burden on the health care system and families alike. Past investigations have reported increased levels of anxiety, depression, and hostility [50], to name a few, for parents of children with ASD. While external interventions may help, the maintenance of these interventions and the aimed improvements are further accelerated or slowed down by the support system that these children have at home. Much of these stresses stem from a lack of an early and continuous understanding of the disorder, the child’s needs, medical and educational provisions, and financial burdens. By identifying potential biomarkers, we can understand the essential mechanisms of the disorder, develop better classification methods based on biological flags, symptoms and characteristics, and contribute to the formation of better diagnostic tools to inform families earlier, since it has been proven that early and timely intensive intervention significantly improves the outcome of the disorder.

In conclusion, considering the effective mechanisms, intrinsic properties, and easy detection and prediction of its progression and regression, cir-ncRNAs have become an attractive prospect for drug targets and diagnostic biomarkers (Figure 6). While further research is still required to suffice the effects of depletion or increased expression of ncRNA on a disorder’s trajectory and severity, their potential as a diagnostic marker and objective identifier of where an individual lies on the spectrum is an exciting and promising area of investigation.

## 6. Patents

A Provisional Patent Application (5600234.00491) has resulted from the work in this manuscript.

## Figures and Tables

**Table 1:** Summary of cohort demographic characteristics.

		Controls	Severe ^1^	Mild ^2^
**N of Cases**		8	35	25
**Gender**				
	Male	4 (50%)	24 (68%)	20 (77%)
	Female	4 (50%)	11 (32%)	5 (23%)
**Age**				
	Median ± SD	10.8 ± 1.8	8.3 ± 2.4	8.5 ± 2.2

^1^ ASD with more symptoms; ^2^ ASD with less symptoms.

**Table 2:** Assays included for measuring spike-ins prior to library preparation.

**Assay Quality Check prior NGS**	**Spike-ins**	**Ct Average**	**Expected Results**
RNA isolation efficiency	UniSp100	31	Delta Ct = 5–7
RNA isolation efficiency	UniSp101	26	
Endogenous control in plasma	miR-103	22	Ct < 40
Endogenous control in plasma	miR-191	18	Ct < 40
Endogenous control in plasma	miR-30c	24	Ct < 40
Hemolysis indicator in plasma	miR-451	25	Delta Ct < 7
Hemolysis indicator in plasma	miR-23	21	
Monitoring presence of inhibitory components	UniSp6	18–20	<2 CTs between any two samples

**Table 3:** Average of the most expressed 20 miRNAs found in the samples.

Mature miRNA	Average
hsa-miR-16-5p	29.5%
hsa-let-7b-5p	14.6%
hsa-let-7a-5p	8.4%
hsa-miR-486-5p	6.6%
hsa-miR-122-5p	4.2%
hsa-let-7f-5p	4.1%
hsa-let-7i-5p	2.6%
hsa-miR-223-3p	2.2%
hsa-miR-142-3p	2.2%
hsa-miR-451a	2.1%
hsa-miR-92a-3p	2%
hsa-miR-21-5p	1.8%
hsa-miR-423-5p	1.6%
hsa-miR-126-3p	1.4%
hsa-miR-26b-5p	1%
hsa-miR-26a-5p	0.8%
hsa-miR-148a-3p	0.7%
hsa-miR-25-3p	0.6%
hsa-miR-101-3p	0.5%
hsa-let-7g-5p	0.4%

**Table 4:** The most differentially expressed miRNAs between ASD vs. Control (Absolute fold change > 2; *p* < 0.05).

miRNA N#	ID	Log_2_ Fold Change	Fold Change	*p*-Value	Symbol
1	hsa-miR-302b-3p	↑12.49	5749.85	1.39 × 10^−8^	miR-291a-3p (and other miRNAs w/seed AAGUGCU)
2	hsa-miR-302a-3p	↑10.27	1233.68	5.32 × 10^−6^	miR-291a-3p (and other miRNAs w/seed AAGUGCU)
3	hsa-miR-302d-3p	↑9.67	814.94	1.21 × 10^−5^	miR-291a-3p (and other miRNAs w/seed AAGUGCU)
4	hsa-miR-144-5p	↑2.35	5.08	4.94 × 10^−6^	miR-144-5p (miRNAs w/seed GAUAUCA)
5	hsa-miR-106a-5p	↑2.18	4.55	2.68 × 10^−3^	miR-17-5p (and other miRNAs w/seed AAAGUGC)
6	hsa-miR-144-3p	↑2.18	4.53	5.97 × 10^−7^	miR-144-3p (miRNAs w/seed ACAGUAU)
7	hsa-miR-122-5p	↑1.95	3.86	8.00 × 10^−5^	miR-122-5p (miRNAs w/seed GGAGUGU)
8	hsa-miR-4306	↑1.82	3.53	3.05 × 10^−3^	miR-185-5p (and other miRNAs w/seed GGAGAGA)
9	hsa-miR-885-3p	↑1.80	3.48	8.12 × 10^−3^	miR-885-3p (miRNAs w/seed GGCAGCG)
10	hsa-miR-182-5p	↑1.80	3.47	4.48 × 10^−5^	miR-182-5p (and other miRNAs w/seed UUGGCAA)
11	hsa-miR-183-5p	↑1.74	3.35	1.86 × 10^−5^	miR-183-5p (miRNAs w/seed AUGGCAC)
12	hsa-miR-584-5p	↓−1.35	−2.54	1.48 × 10^−3^	miR-584-5p (and other miRNAs w/seed UAUGGUU)
13	hsa-miR-99a-5p	↓−1.37	−2.58	2.35 × 10^−2^	miR-100-5p (and other miRNAs w/seed ACCCGUA)
14	hsa-miR-4433b-5p	↓−1.39	−2.62	4.60 × 10^−3^	miR-4433b-5p (miRNAs w/seed UGUCCCA)
15	hsa-miR-127-3p	↓−1.39	−2.63	8.64 × 10^−3^	miR-127-3p (miRNAs w/seed CGGAUCC)
16	hsa-miR-12136	↓−1.43	−2.69	1.80 × 10^−2^	miR-12136 (miRNAs w/seed AAAAAGU)
17	hsa-miR-6852-5p	↓−1.45	−2.73	2.65 × 10^−2^	miR-6852-5p (and other miRNAs w/seed CCUGGGG)
18	hsa-miR-744-5p	↓−1.48	−2.78	5.27 × 10^−3^	miR-744-5p (and other miRNAs w/seed GCGGGGC)
19	hsa-miR-134-5p	↓−1.48	−2.80	9.93 × 10^−3^	miR-3118 (and other miRNAs w/seed GUGACUG)
20	hsa-miR-23a-5p	↓−1.67	−3.18	1.06 × 10^−2^	miR-23a-5p (and other miRNAs w/seed GGGUUCC)
21	hsa-miR-221-3p	↓−1.67	−3.19	7.85 × 10^−4^	miR-221-3p (and other miRNAs w/seed GCUACAU)
22	hsa-miR-4433b-3p	↓−1.72	−3.28	2.22 × 10^−2^	miR-4433b-3p (miRNAs w/seed AGGAGUG)

↓ Downregulated; ↑ Upregulated.

**Table 5:** Differentially expressed piRNAs between ASD vs. Control (Absolute fold change > 2; *p* < 0.05).

	Name	Max Group Mean	Log_2_ Fold Change	Fold Change	*p*-Value
1	piR-hsa-1282	116	↑5.28	38.87	9.4 × 10^−6^
2	piR-hsa-12790	95	↑4.28	19.48	2.7 × 10^−4^
3	piR-hsa-23326	63	↑3.79	13.81	2.3 × 10^−4^
4	piR-hsa-1207	1447	↑3.55	11.75	1.6 × 10^−4^
5	piR-hsa-28131	1448	↑3.52	11.51	1.3 × 10^−4^
6	piR-hsa-6463	60	↑3.36	10.30	3.9 × 10^−4^
7	piR-hsa-1242	69	↑3.15	8.90	9.8 × 10^−5^
8	piR-hsa-27493	512	↑3.10	8.60	6.7 × 10^−4^
9	piR-hsa-27620	69	↑3.06	8.33	1.8 × 10^−4^
10	piR-hsa-27621	80	↑2.99	7.97	8.7 × 10^−4^
11	piR-hsa-27140	50	↑2.99	7.94	1.2 × 10^−3^
12	piR-hsa-1243	81	↑2.59	6.00	1.4 × 10^−3^
13	piR-hsa-23533	165	↑2.49	5.61	2.3 × 10^−4^
14	piR-hsa-23248	70	↑2.19	4.56	1.2 × 10^−2^
15	piR-hsa-28876	205	↑2.11	4.33	8.9 × 10^−3^
16	piR-hsa-27622	93	↑2.08	4.23	6.9 × 10^−3^
17	piR-hsa-1177	541	↑1.99	3.98	9.0 × 10^−4^
18	piR-hsa-28190	53	↑1.97	3.92	4.5 × 10^−3^
19	piR-hsa-5937	237	↑1.87	3.66	1.3 × 10^−2^
20	piR-hsa-24672	239	↑1.78	3.45	1.6 × 10^−2^
21	piR-hsa-28877	285	↑1.36	2.58	3.7 × 10^−2^
22	piR-hsa-28390	75	↓−1.05	−2.08	1.1 × 10^−2^
23	piR-hsa-32235	63	↓−1.08	−2.12	4.2 × 10^−2^
24	piR-hsa-28374	108	↓−1.14	−2.20	2.2 × 10^−2^
25	piR-hsa-32195	745	↓−1.32	−2.50	3.6 × 10^−2^
26	piR-hsa-23210	3502	↓−1.41	−2.66	2.8 × 10^−2^
27	piR-hsa-32238	274	↓−1.42	−2.68	3.8 × 10^−2^
28	piR-hsa-27731	266	↓−1.48	−2.79	4.0 × 10^−2^
29	piR-hsa-27730	110	↓−1.68	−3.21	2.4 × 10^−2^
30	piR-hsa-325	55	↓−1.68	−3.22	1.1 × 10^−2^
31	piR-hsa-27729	100	↓−1.69	−3.24	2.1 × 10^−2^
32	piR-hsa-1849	204	↓−1.74	−3.34	2.1 × 10^−2^
33	piR-hsa-32158	205	↓−1.88	−3.70	2.8 × 10^−3^
34	piR-hsa-23209	71,482	↓−1.99	−3.97	1.4 × 10^−3^
35	piR-hsa-32182	6232	↓−1.99	−3.98	1.3 × 10^−3^
36	piR-hsa-32167	4377	↓−2.08	−4.24	6.7 × 10^−4^
37	piR-hsa-32159	51380	↓−2.09	−4.27	7.7 × 10^−4^

↓ Downregulated; ↑ Upregulated.

**Table 6:** Differentially expressed YRNAs and snoRNAs between ASD vs. Control (Absolute fold change > 2; *p* < 0.05).

ncRNA Type	Name	Chromosome	Region	Max Group Mean	Log_2_ Fold Change	Fold Change	*p*-Value
**Y-RNA**	RNY4P36	17	c (76703632..76703727)	63	↓−1.46	−2.74	4.72 × 10^−2^
	RNY4P6	11	117015897..117015992	302	↓−1.80	−3.49	1.63 × 10^−2^
	RNY4	7	148963315..148963410	337946	↓−1.86	−3.65	9.88 × 10^−3^
	RNY4P25	1	151439000..151439095	990	↓−1.89	−3.72	9.83 × 10^−3^
	RNY4P18	9	c (111097325..111097413)	149	↓−1.92	−3.79	6.03 × 10^−3^
**snoRNA**	SNORD3C	17	c (19189665..19190245)	327	↑3.58	11.98	3.66 × 10^−4^
	SNORD69	3	52692736..52692812	351	↑3.45	10.96	1.13 × 10^−2^
	SNORD51	2	206161878..206161957	289	↑3.41	10.65	5.95 × 10^−3^
	SNORD10	17	7576811..7576952	265	↑3.19	9.14	1.16 × 10^−3^
	SNORD22	11	c (62852910..62853035)	94	↑3.14	8.85	2.60 × 10^−2^
	SNORD24	9	133349396..133349470	135	↑2.88	7.37	3.15 × 10^−2^
	SNORD102	13	27255064..27255135	828	↑2.31	4.96	8.83 × 10^−3^
	SNORD3A	17	19188016..19188714	1165	↑2.15	4.45	2.75 × 10^−2^
	SNORD26	11	c (62855292..62855366)	1974	↑2.07	4.19	2.17 × 10^−2^
	SNORA63B	3	186786323..186786445	149	↓−1.52	−2.87	4.70 × 10^−2^
	SNORD65	17	16441226..16441298	89	↓−1.65	−3.15	3.31 × 10^−2^
	SNORA51	20	2655067..2655198	341	↓−1.73	−3.32	2.90 × 10^−2^
	SNORD57	20	2656939..2657010	119	↓−2.67	−6.38	6.23 × 10^−4^

c: Complement ↓ Downregulated; ↑ Upregulated.

**Table 7:** The most differentially expressed miRNAs between individuals that expressed more symptoms (severe) and less symptoms (mild) (Absolute fold change > 2; *p* < 0.05).

miRNA N#	ID	Log_2_ Fold Change	Fold Change	*p*-Value	Symbol
1	hsa-miR-302a-5p	↑8.70	416.11	2.4 × 10^−14^	miR-302a-5p (miRNAs w/seed CUUAAAC)
2	hsa-miR-302c-3p	↑7.51	182.42	4.6 × 10^−14^	miR-291a-3p (and other miRNAs w/seed AAGUGCU)
3	hsa-miR-302a-3p	↑7.24	152.01	4.9 × 10^−14^	miR-291a-3p (and other miRNAs w/seed AAGUGCU)
4	hsa-miR-302d-3p	↑7.23	150.37	8.3 × 10^−15^	miR-291a-3p (and other miRNAs w/seed AAGUGCU)
5	hsa-miR-302b-3p	↑6.95	123.89	2.2 × 10^−16^	miR-291a-3p (and other miRNAs w/seed AAGUGCU)
6	hsa-miR-302c-5p	↑6.56	94.36	1.5 × 10^−9^	miR-302c-5p (miRNAs w/seed UUAACAU)
7	hsa-miR-135b-5p	↑5.49	44.93	2.2 × 10^−7^	miR-135a-5p (and other miRNAs w/seed AUGGCUU)
8	hsa-miR-373-3p	↑5.02	32.39	1.8 × 10^−5^	miR-291a-3p (and other miRNAs w/seed AAGUGCU)
9	hsa-miR-372-3p	↑4.75	26.90	8.5 × 10^−7^	miR-291a-3p (and other miRNAs w/seed AAGUGCU)
10	hsa-miR-187-3p	↑4.37	20.66	6.0 × 10^−6^	miR-187-3p (miRNAs w/seed CGUGUCU)
11	hsa-miR-302b-5p	↑4.36	20.48	1.0 × 10^−4^	miR-302b-5p (and other miRNAs w/seed CUUUAAC)
12	hsa-miR-4648	↓−1.79	−3.47	4.4 × 10^−2^	miR-4648 (miRNAs w/seed GUGGGAC)
13	hsa-miR-450a-2-3p	↓−1.91	−3.76	1.2 × 10^−2^	miR-450b-3p (and other miRNAs w/seed UUGGGGA)
14	hsa-miR-4745-5p	↓−1.95	−3.85	4.9 × 10^−2^	miR-4481 (and other miRNAs w/seed GAGUGGG)
15	hsa-miR-184	↓−2.11	−4.32	6.0 × 10^−3^	miR-184 (and other miRNAs w/seed GGACGGA)
16	hsa-miR-219a-5p	↓−2.12	−4.36	1.8 × 10^−2^	miR-219a-5p (and other miRNAs w/seed GAUUGUC)
17	hsa-miR-128-1-5p	↓−2.13	−4.39	1.9 × 10^−2^	miR-128-1-5p (and other miRNAs w/seed GGGGCCG)
18	hsa-miR-6516-5p	↓−2.22	−4.67	7.0 × 10^−3^	miR-6516-5p (miRNAs w/seed UUGCAGU)
19	hsa-miR-5189-5p	↓−2.52	−5.74	2.4 × 10^−14^	miR-1285-3p (and other miRNAs w/seed CUGGGCA)
20	hsa-miR-378g	↓−2.53	−5.79	4.6 × 10^−14^	miR-378g (miRNAs w/seed CUGGGCU)
21	hsa-let-7f-2-3p	↓−2.79	−6.94	4.9 × 10^−14^	let-7f-2-3p (and other miRNAs w/seed UAUACAG)
22	hsa-miR-6509-5p	↓−3.05	−8.33	8.3 × 10^−15^	miR-6509-5p (miRNAs w/seed UUAGGUA)

↓ Downregulated; ↑ Upregulated.

**Table 8:** Differentially expressed piRNAs (N = 29) between individuals with severe symptoms vs. mild symptoms (Absolute fold change > 2; *p* < 0.05).

	Name	Max Group Mean	Log_2_ Fold Change	Fold Change	*p*-Value
1	piR-hsa-22380	405	↑4.63	24.73	8.80 × 10^−8^
2	piR-hsa-28131	226	↑3.69	12.88	8.80 × 10^−8^
3	piR-hsa-27134	238	↑3.65	12.59	8.80 × 10^−8^
4	piR-hsa-27138	102	↑3.03	8.20	8.80 × 10^−8^
5	piR-hsa-27619	85	↑2.87	7.32	8.80 × 10^−8^
6	piR-hsa-28877	611	↑2.70	6.51	3.30 × 10^−8^
7	piR-hsa-28190	115	↑2.66	6.34	5.40 × 10^−7^
8	piR-hsa-28876	108	↑2.65	6.26	5.40 × 10^−7^
9	piR-hsa-5937	146	↑2.63	6.20	1.00 × 10^−2^
10	piR-hsa-27621	98	↑2.58	5.98	1.00 × 10^−2^
11	piR-hsa-24683	65	↑2.53	5.77	1.00 × 10^−2^
12	piR-hsa-26508	61	↑2.22	4.67	2.00 × 10^−2^
13	piR-hsa-27140	142	↑2.17	4.51	2.00 × 10^−2^
14	piR-hsa-6463	59	↑2.14	4.41	1.00 × 10^−2^
15	piR-hsa-27620	67	↑2.09	4.26	2.00 × 10^−2^
16	piR-hsa-32207	73	↑2.08	4.23	2.00 × 10^−2^
17	piR-hsa-24672	80	↑2.05	4.14	3.00 × 10^−2^
18	piR-hsa-32221	251	↑2.00	3.99	3.00 × 10^−2^
19	piR-hsa-1242	102	↑1.92	3.78	2.00 × 10^−2^
20	piR-hsa-32184	683	↑1.63	3.10	4.00 × 10^−2^
21	piR-hsa-1243	210	↑1.52	2.87	3.00 × 10^−2^
22	piR-hsa-27493	314	↑1.42	2.68	4.00 × 10^−2^
23	piR-hsa-12790	224	↓−1.24	−2.36	2.00 × 10^−2^
24	piR-hsa-1282	27312	↓−1.47	−2.76	1.00 × 10^−2^
25	piR-hsa-23679	373	↓−1.47	−2.77	1.00 × 10^−2^
26	piR-hsa-27622	49	↓−2.04	−4.10	2.00 × 10^−2^
27	piR-hsa-32798	151	↓−2.34	−5.05	1.00 × 10^−2^
28	piR-hsa-32175	80	↓−3.38	−10.44	1.00 × 10^−2^
29	piR-hsa-27623	154	↓−3.70	−12.98	1.00 × 10^−2^

↓ Downregulated; ↑ Upregulated.

**Table 9:** Differentially expressed Y-RNAs (N = 4) and snoRNAs (N = 23) between individuals with more symptoms (severe) vs. less symptoms (mild) (Absolute fold change > 2; *p* < 0.05).

ncRNA Type	Name	Chromosome	Region	Max Group Mean	Log_2_ Fold Change	Fold Change	*p*-Value
Y-RNA	RNY4P29	13	58527655..58527751	102.46	↑3.03	8.20	1.00 × 10^−2^
	RNY3P1	5	79170234..79170335	224.19	↓−1.24	−2.36	2.00 × 10^−2^
	RNY3	7	148983755..148983856	27,311.52	↓−1.47	−2.76	1.00 × 10^−2^
	RNY4P28	13	60187738..60187830	372.93	↓−1.47	−2.77	1.00 × 10^−2^
snoRNA	SNORA69	X	119787353..119787484	405.03	↑4.63	24.73	1.00 × 10^−2^
	SNORA41	2	206162228..206162359	225.52	↑3.69	12.88	1.00 × 10^−2^
	SNORA46	16	58548499..58548633	238.04	↑3.65	12.59	1.00 × 10^−2^
	SNORA13	5	112161485..112161617	107.75	↑2.65	6.26	1.00 × 10^−2^
	SNORD60	16	2155023..2155105	146.18	↑2.63	6.20	1.00 × 10^−2^
	SNORA28	14	103337849..103337974	97.82	↑2.58	5.98	1.00 × 10^−2^
	SNORA52	11	811681..811814	65.41	↑2.53	5.77	1.00 × 10^−2^
	SNORA74D	5	139276180..139276320	60.87	↑2.22	4.67	2.00 × 10^−2^
	SNORD72	5	40832656..40832735	142.01	↑2.17	4.51	2.00 × 10^−2^
	SNORA73A	1	28507366..28507571	58.64	↑2.14	4.41	1.00 × 10^−2^
	SNORD111B	16	70529509..70529588	67.19	↑2.09	4.26	2.00 × 10^−2^
	SNORA62	3	39411054..39411206	73.02	↑2.08	4.23	2.00 × 10^−2^
	SNORD7	17	35573657..35573753	79.74	↑2.05	4.14	3.00 × 10^−2^
	SNORA26	4	52713249..52713370	250.51	↑2.00	3.99	1.00 × 10^−2^
	SNORD83A	22	39315213..39315307	102.28	↑1.92	3.78	2.00 × 10^−2^
	SNORA63	3	186787300..186787431	29.43	↑1.72	3.30	5.00 × 10^−2^
	SNORD101	6	132815307..132815379	682.64	↑1.63	3.10	4.00 × 10^−2^
	SNORD36B	9	133350095..133350168	210.17	↑1.52	2.87	3.00 × 10^−2^
	SNORD2	3	186784796..186784864	313.60	↑1.42	2.68	4.00 × 10^−2^
	SNORD46	1	44776490..44776593	42.20	↓−1.85	−3.61	2.00 × 10^−2^
	SNORD43	22	39319050..39319113	150.66	↓−2.34	−5.05	1.00 × 10^−2^
	SNORD73A	4	151103827..151103891	79.96	↓−3.38	−10.44	1.00 × 10^−2^
	SNORD42A	17	28723429..28723492	153.51	↓−3.70	−12.98	1.00 × 10^−2^

↓ Downregulated; ↑ Upregulated.

**Table 10:** Ingenuity Pathways Analysis (IPA) summary.

		Name	*p*-Value Range
**ASD vs. Control**	**Diseases and Disorders**	Organismal Injury and Abnormalities	4.82 × 10^−2^–1.70 × 10^−18^
		Reproductive System Disease	3.05 × 10^−2^–7.82.45 × 10^−18^
		Psychological Disorders	4.25 × 10^−2^–1.38 × 10^−12^
		Neurological Disease	3.07 × 10^−2^–1.38 × 10^−12^
	**Molecular and Cellular Functions**	Cellular Growth and Proliferation	4.82 × 10^−2^–7.4 × 10^−10^
		Celillar Movement	4.28 × 10^−2^–4.9 × 10^−7^
		Cell Cycle	4.82 × 10^−2^–1.08 × 10^−5^
		Cel Death and Survival	4.29 × 10^−2^–8.95 × 10^−5^
**Severe vs. Mild**	**Diseases and Disorders**	Organismal Injury and Abnormalities	4.97 × 10^−2^–1.45 × 10^−25^
		Reproductive System Disease	4.74 × 10^−2^–1.45 × 10^−25^
		Psychological Disorders	2.70 × 10^−3^–3.09 × 10^−15^
		Neurological Disease	4.72 × 10^−2^–4.38 × 10^−15^
		Cellular Movement	4.04 × 10^−2^–2.17 × 10^−6^
	**Molecular and Cellular Functions**	Cell Cycle	3.66 × 10^−2^–2.62 × 10^−11^
		Cellular Development	4.72 × 10^−2^–3.93 × 10^−11^
		Cellular Growth and Proliferation	3.66 × 10^−2^–3.48 × 10^−9^

## Supplementary Materials

The following are available online at https://www.mdpi.com/article/10.3390/ijms22126549/s1, Supplementary Tables S1–S6.
