# Supplementary material for: Circulating Non-Coding RNAs as a Signature of Autism Spectrum Disorder Symptomatology
Source: Int J Mol Sci. 2021 Jun 18;22(12):6549. doi: 10.3390/ijms22126549 (PMC8235321; doi:10.3390/ijms22126549)
Supplement: Supplementary file 1 [file ijms-22-06549-s001.zip › ijms-1225505-SI.pdf]

Supplementary Tables

Supplementary Table S1: miRNAs differential expression analysis: ASD cases *vs.* Controls

N#	ID	Log2 Fold change	Fold Change	p-value	Symbol
**1**	**hsa-miR-302b-3p**	**12.49**	**5749.85**	**1.39E-08**	**miR-291a-3p (and other miRNAs w/seed AAGUGCU)**
**2**	**hsa-miR-302a-3p**	**10.27**	**1233.68**	**5.32E-06**	**miR-291a-3p (and other miRNAs w/seed AAGUGCU)**
**3**	**hsa-miR-302d-3p**	**9.67**	**814.94**	**1.21E-05**	**miR-291a-3p (and other miRNAs w/seed AAGUGCU)**
**4**	**hsa-miR-144-5p**	**2.35**	**5.08**	**4.94E-06**	**miR-144-5p (miRNAs w/seed GAUAUCA)**
**5**	**hsa-miR-106a-5p**	**2.18**	**4.55**	**2.68E-03**	**miR-17-5p (and other miRNAs w/seed AAAGUGC)**
**6**	**hsa-miR-144-3p**	**2.18**	**4.53**	**5.97E-07**	**miR-144-3p (miRNAs w/seed ACAGUAU)**
**7**	**hsa-miR-122-5p**	**1.95**	**3.86**	**8.00E-05**	**miR-122-5p (miRNAs w/seed GGAGUGU)**
**8**	**hsa-miR-4306**	**1.82**	**3.53**	**3.05E-03**	**miR-185-5p (and other miRNAs w/seed GGAGAGA)**
**9**	**hsa-miR-885-3p**	**1.80**	**3.48**	**8.12E-03**	**miR-885-3p (miRNAs w/seed GGCAGCG)**
**10**	**hsa-miR-182-5p**	**1.80**	**3.47**	**4.48E-05**	**miR-182-5p (and other miRNAs w/seed UUGGCAA)**
**11**	**hsa-miR-183-5p**	**1.74**	**3.35**	**1.86E-05**	**miR-183-5p (miRNAs w/seed AUGGCAC)**
**12**	**hsa-miR-874-3p**	**1.56**	**2.94**	**2.26E-03**	**miR-874-3p (miRNAs w/seed UGCCCUG)**
**13**	**hsa-miR-141-3p**	**1.54**	**2.90**	**1.39E-04**	**miR-141-3p (and other miRNAs w/seed AACACUG)**
**14**	**hsa-miR-1180-3p**	**1.52**	**2.87**	**5.19E-04**	**miR-1180-3p (miRNAs w/seed UUCCGGC)**
**15**	**hsa-miR-29c-3p**	**1.49**	**2.80**	**1.55E-05**	**miR-29b-3p (and other miRNAs w/seed AGCACCA)**
**16**	**hsa-miR-96-5p**	**1.47**	**2.78**	**1.18E-03**	**miR-96-5p (and other miRNAs w/seed UUGGCAC)**
**17**	**hsa-miR-1294**	**1.45**	**2.73**	**1.91E-03**	**miR-1294 (and other miRNAs w/seed GUGAGGU)**
**18**	**hsa-miR-3613-5p**	**1.42**	**2.67**	**1.12E-04**	**miR-3613-5p (miRNAs w/seed GUUGUAC)**
**19**	**hsa-miR-101-3p**	**1.39**	**2.62**	**3.15E-04**	**miR-101-3p (and other miRNAs w/seed ACAGUAC)**
**20**	**hsa-miR-4732-5p**	**1.37**	**2.59**	**1.08E-02**	**miR-4732-5p (miRNAs w/seed GUAGAGC)**
**21**	**hsa-miR-193b-5p**	**1.34**	**2.52**	**1.12E-02**	**miR-193b-5p (miRNAs w/seed GGGGUUU)**
**22**	**hsa-miR-196a-5p**	**1.30**	**2.46**	**1.54E-02**	**miR-196a-5p (and other miRNAs w/seed AGGUAGU)**
**23**	**hsa-miR-106b-5p**	**1.28**	**2.43**	**1.63E-03**	**miR-17-5p (and other miRNAs w/seed AAAGUGC)**
**24**	**hsa-miR-4742-3p**	**1.28**	**2.42**	**1.53E-02**	**miR-4742-3p (miRNAs w/seed CUGUAUU)**
**25**	**hsa-miR-29b-3p**	**1.26**	**2.40**	**3.51E-04**	**miR-29b-3p (and other miRNAs w/seed AGCACCA)**
**26**	**hsa-miR-199b-5p**	**1.22**	**2.33**	**3.58E-02**	**miR-199a-5p (and other miRNAs w/seed CCAGUGU)**
**27**	**hsa-miR-636**	**1.19**	**2.29**	**2.49E-02**	**miR-636 (miRNAs w/seed GUGCUUG)**
**28**	**hsa-miR-194-5p**	**1.18**	**2.27**	**2.81E-03**	**miR-194-5p (miRNAs w/seed GUAACAG)**
**29**	**hsa-miR-542-3p**	**1.16**	**2.23**	**3.27E-02**	**miR-542-3p (miRNAs w/seed GUGACAG)**
**30**	**hsa-miR-660-5p**	**1.14**	**2.20**	**6.28E-04**	**miR-660-5p (and other miRNAs w/seed ACCCAUU)**
**31**	**hsa-miR-1255b-5p**	**1.11**	**2.15**	**1.76E-02**	**miR-1255b-5p (and other miRNAs w/seed GGAUGAG)**
**32**	**hsa-miR-150-3p**	**1.10**	**2.15**	**1.21E-02**	**miR-150-3p (miRNAs w/seed UGGUACA)**
**33**	**hsa-miR-7-5p**	**1.10**	**2.14**	**8.21E-04**	**miR-7a-5p (and other miRNAs w/seed GGAAGAC)**
**34**	**hsa-miR-20b-5p**	**1.10**	**2.14**	**9.78E-03**	**miR-17-5p (and other miRNAs w/seed AAAGUGC)**
**35**	**hsa-miR-19a-3p**	**1.07**	**2.09**	**2.14E-03**	**miR-19b-3p (and other miRNAs w/seed GUGCAAA)**
**36**	**hsa-miR-148b-5p**	**1.06**	**2.09**	**1.20E-02**	**miR-148b-5p (and other miRNAs w/seed AGUUCUG)**
**37**	**hsa-miR-15a-5p**	**1.06**	**2.09**	**8.83E-04**	**miR-16-5p (and other miRNAs w/seed AGCAGCA)**
**38**	**hsa-miR-192-5p**	**1.06**	**2.08**	**7.40E-03**	**miR-192-5p (and other miRNAs w/seed UGACCUA)**
**39**	**hsa-miR-375-3p**	**1.05**	**2.07**	**2.79E-02**	**miR-375-3p (miRNAs w/seed UUGUUCG)**
**40**	**hsa-let-7b-5p**	**1.05**	**2.07**	**5.05E-03**	**let-7a-5p (and other miRNAs w/seed GAGGUAG)**
41	hsa-miR-28-3p	-1.03	-2.04	1.19E-02	miR-28-3p (and other miRNAs w/seed ACUAGAU)
42	hsa-miR-3928-3p	-1.03	-2.05	4.65E-02	miR-3928-3p (miRNAs w/seed GAGGAAC)
43	hsa-miR-328-3p	-1.06	-2.08	1.88E-02	miR-328-3p (and other miRNAs w/seed UGGCCCU)
44	hsa-miR-5189-5p	-1.06	-2.09	3.92E-02	miR-1285-3p (and other miRNAs w/seed CUGGGCA)
45	hsa-miR-2355-3p	-1.08	-2.11	2.64E-02	miR-2355-3p (miRNAs w/seed UUGUCCU)
46	hsa-miR-335-5p	-1.09	-2.13	7.65E-03	miR-335-5p (and other miRNAs w/seed CAAGAGC)
47	hsa-miR-1301-3p	-1.11	-2.16	1.56E-02	miR-1301-3p (and other miRNAs w/seed UGCAGCU)

48	hsa-miR-485-3p	-1.14	-2.20	2.09E-02	miR-485-3p (and other miRNAs w/seed UCAUACA)
49	hsa-miR-1908-5p	-1.20	-2.30	1.59E-02	miR-10396b-5p (and other miRNAs w/seed GGCGGGG)
50	hsa-miR-224-5p	-1.22	-2.32	5.09E-03	miR-125b-2-3p (and other miRNAs w/seed CAAGUCA)
51	hsa-miR-4446-3p	-1.27	-2.42	3.78E-02	miR-4446-3p (miRNAs w/seed AGGGCUG)
52	hsa-miR-485-5p	-1.31	-2.48	2.44E-02	miR-485-5p (and other miRNAs w/seed GAGGCUG)
53	hsa-miR-584-5p	-1.35	-2.54	1.48E-03	miR-584-5p (and other miRNAs w/seed UAUGGUU)
54	hsa-miR-99a-5p	-1.37	-2.58	2.35E-02	miR-100-5p (and other miRNAs w/seed ACCCGUA)
55	hsa-miR-4433b-5p	-1.39	-2.62	4.60E-03	miR-4433b-5p (miRNAs w/seed UGUCCCA)
56	hsa-miR-127-3p	-1.39	-2.63	8.64E-03	miR-127-3p (miRNAs w/seed CGGAUCC)
57	hsa-miR-12136	-1.43	-2.69	1.80E-02	miR-12136 (miRNAs w/seed AAAAAGU)
58	hsa-miR-6852-5p	-1.45	-2.73	2.65E-02	miR-6852-5p (and other miRNAs w/seed CCUGGGG)
59	hsa-miR-744-5p	-1.48	-2.78	5.27E-03	miR-744-5p (and other miRNAs w/seed GCGGGGC)
60	hsa-miR-134-5p	-1.48	-2.80	9.93E-03	miR-3118 (and other miRNAs w/seed GUGACUG)
61	hsa-miR-23a-5p	-1.67	-3.18	1.06E-02	miR-23a-5p (and other miRNAs w/seed GGGUUCC)
62	hsa-miR-221-3p	-1.67	-3.19	7.85E-04	miR-221-3p (and other miRNAs w/seed GCUACAU)
63	hsa-miR-4433b-3p	-1.72	-3.28	2.22E-02	miR-4433b-3p (miRNAs w/seed AGGAGUG)

Supplementary Table S2: Other ncRNAs differential expression analysis: ASD cases *vs.* Controls

N#	Name	Chromosome	Region	Max group mean	Log_2 fold change	Fold change	P-value
1	MT-ND1	MT	3307..4262	174	-1.53	-2.88	4.67E-02
2	MT-RNR1	MT	648..1601	3749	-1.62	-3.08	2.13E-02
3	MT-ND5	MT	12337..14148	113	-1.65	-3.13	4.66E-02
4	MT-ND3	MT	10059..10404	132	-1.74	-3.34	4.90E-02
5	MT-TW	MT	5512..5579	981	-1.74	-3.35	4.22E-02
6	MT-TE	MT	c(14674..14742)	187331	-1.79	-3.45	4.05E-02
7	MT-RNR2	MT	1671..3229	16216	-1.81	-3.51	3.67E-02
8	MT-TS2	MT	12207..12265	149289	-1.82	-3.54	4.97E-02
9	MT-TS1	MT	c(7446..7514)	19111	-1.83	-3.54	3.17E-02
10	MT-TG	MT	9991..10058	8975	-1.85	-3.61	3.36E-02
11	MT-TT	MT	15888..15953	37231	-1.90	-3.72	3.26E-02
12	MT-TV	MT	1602..1670	265677	-1.92	-3.77	3.14E-02
13	MT-TH	MT	12138..12206	863077	-1.93	-3.80	4.03E-02
14	MT-CO3	MT	9207..9990	906	-2.05	-4.13	1.95E-02
15	MT-TK	MT	8295..8364	15625	-2.09	-4.26	1.48E-02
16	MT-TI	MT	4263..4331	18263	-2.14	-4.42	1.84E-02
17	MT-TP	MT	c(15956..16023)	16194	-2.17	-4.49	5.29E-03
18	MT-TQ	MT	c(4329..4400)	55467	-2.18	-4.52	9.23E-03
19	MT-TY	MT	c(5826..5891)	57752	-2.25	-4.76	3.32E-03
20	MT-TN	MT	c(5657..5729)	15641	-2.25	-4.76	1.07E-02
21	MT-TL2	MT	12266..12336	28417	-2.28	-4.85	7.57E-03
22	MT-TL1	MT	3230..3304	9813	-2.31	-4.96	3.19E-03
23	MT-TD	MT	7518..7585	53034	-2.44	-5.41	2.64E-03
24	MT-TC	MT	c(5761..5826)	15881	-2.45	-5.47	2.12E-03
25	MT-TR	MT	10405..10469	14563	-2.46	-5.49	2.37E-03
**26**	**AC104248.1**	**8**	**c(108871128..109063417)**	**1093**	**7.62**	**196.82**	**1.59E-12**
**27**	**LY75-CD302**	**2**	**c(159771851..159904710)**	**103**	**6.66**	**101.26**	**2.67E-08**
**28**	**AC020661.4**	**15**	**c(41016990..41018843)**	**92**	**5.75**	**53.78**	**1.46E-05**
**29**	**AC099681.3**	**7**	**55592074..55593193**	**80**	**4.50**	**22.66**	**3.63E-04**
**30**	**AC087235.1**	**12**	**c(23181334..23251499)**	**52**	**3.82**	**14.11**	**1.06E-05**
**31**	**AL357052.1**	**6**	**c(19290319..19321021)**	**614**	**3.37**	**10.33**	**1.84E-07**
**32**	**AC040170.1**	**16**	**c(86275429..86275523)**	**195**	**2.99**	**7.97**	**3.76E-02**

**33**	**AC090227.1**	**18**	**49814361..49814443**	**117**	**2.91**	**7.52**	**3.80E-02**
**34**	**AP001591.1**	**11**	**63495484..63500610**	**50**	**2.88**	**7.37**	**3.39E-02**
**35**	**AC013267.2**	**2**	**c(25008845..25008946)**	**675**	**2.81**	**7.04**	**7.96E-03**
**36**	**LINC02739**	**11**	**c(59560298..59566283)**	**903**	**2.11**	**4.32**	**2.78E-03**
37	AC079449.1	2	c(121603073..121603164)	195	-1.36	-2.56	4.79E-02
38	AC209007.2	1	c(31503631..31503744)	67	-1.46	-2.74	4.87E-02
39	AC026634.1	18	23456371..23456467	57	-1.56	-2.94	2.12E-02
40	AP003392.15	11	119018944..119019012	405	-1.57	-2.98	3.23E-02
41	AC012572.1	18	c(78544095..78544188)	190	-1.58	-2.99	4.11E-03
42	AC104066.1	4	52786537..52786638	59	-1.58	-3.00	3.66E-02
43	AL137009.1	6	119057880..119057974	15563	-1.60	-3.02	4.35E-02
44	AC000114.1	X	112661657..112661768	79	-1.60	-3.04	4.57E-03
45	AL512288.1	1	c(27938875..27960193)	263	-1.64	-3.11	4.47E-02
46	AL139406.1	10	c(88585638..88585733)	498	-1.65	-3.15	1.22E-02
47	AC217773.1	17	c(46215070..46215171)	75	-1.70	-3.25	8.88E-03
48	AC020987.1	8	c(70780914..70781008)	110	-1.72	-3.28	9.63E-03
49	AC005037.2	2	c(200863152..200863247)	337	-1.76	-3.39	4.45E-02
50	NR2F2-AS1	15	c(96110040..96327361)	1058	-1.79	-3.47	2.45E-02
51	AP004609.2	11	118970498..118970595	128	-1.82	-3.52	1.47E-02
52	AL627171.4	14	c(49861176..49864326)	80	-1.82	-3.53	1.48E-02
53	LINC01962	5	c(181178821..181191852)	424	-1.91	-3.76	9.90E-03
54	AL080243.1	22	c(41065554..41065646)	61	-1.96	-3.90	3.80E-04
55	AL390766.1	10	c(34201979..34202074)	6285	-1.98	-3.94	2.15E-02
56	AC060812.2	11	c(3663815..3663911)	14092	-2.15	-4.44	9.12E-04
57	AC107939.1	11	c(33004250..33004345)	2873	-2.34	-5.07	7.20E-04
58	AC130462.1	16	c(68089449..68089543)	70	-2.43	-5.38	9.93E-05
59	AL359839.1	1	c(44153385..44153480)	426	-2.51	-5.70	1.33E-05
60	AC011603.1	12	c(49132853..49132947)	371	-2.54	-5.80	1.09E-04
61	LINC01714	1	8201518..8215210	151	-2.61	-6.10	2.64E-03
62	AL357075.2	6	154477982..154479593	70	-3.16	-8.97	1.05E-02
63	AC096551.1	7	c(115833273..115833373)	69	-3.17	-8.99	2.24E-06
64	AC023356.1	15	c(43863893..43864005)	208	-4.64	-24.94	2.67E-09
65	AC026353.1	3	c(167866500..167950292)	80	-5.52	-45.79	1.03E-09
66	AC010443.1	5	c(144087..144197)	441	-5.76	-54.15	4.14E-12

c: complement

Supplementary Table S3: miRNAs differential expression analysis: severe symptoms *vs.* mild symptoms cases

N#	ID	Log_2 Fold change	Fold Change	p-value	Symbol
**1**	**hsa-miR-302a-5p**	**8.70**	**416.11**	**2.36E-14**	**miR-302a-5p (miRNAs w/seed CUUAAAC)**
**2**	**hsa-miR-302c-3p**	**7.51**	**182.42**	**4.60E-14**	**miR-291a-3p (and other miRNAs w/seed AAGUGCU)**
**3**	**hsa-miR-302a-3p**	**7.24**	**152.01**	**4.85E-14**	**miR-291a-3p (and other miRNAs w/seed AAGUGCU)**
**4**	**hsa-miR-302d-3p**	**7.23**	**150.37**	**8.33E-15**	**miR-291a-3p (and other miRNAs w/seed AAGUGCU)**
**5**	**hsa-miR-302b-3p**	**6.95**	**123.89**	**2.22E-16**	**miR-291a-3p (and other miRNAs w/seed AAGUGCU)**
**6**	**hsa-miR-302c-5p**	**6.56**	**94.36**	**1.47E-09**	**miR-302c-5p (miRNAs w/seed UUAACAU)**
**7**	**hsa-miR-135b-5p**	**5.49**	**44.93**	**2.20E-07**	**miR-135a-5p (and other miRNAs w/seed AUGGCUU)**
**8**	**hsa-miR-373-3p**	**5.02**	**32.39**	**1.76E-05**	**miR-291a-3p (and other miRNAs w/seed AAGUGCU)**
**9**	**hsa-miR-372-3p**	**4.75**	**26.90**	**8.48E-07**	**miR-291a-3p (and other miRNAs w/seed AAGUGCU)**
**10**	**hsa-miR-187-3p**	**4.37**	**20.66**	**6.04E-06**	**miR-187-3p (miRNAs w/seed CGUGUCU)**
**11**	**hsa-miR-302b-5p**	**4.36**	**20.48**	**1.00E-04**	**miR-302b-5p (and other miRNAs w/seed CUUUAAC)**
**12**	**hsa-miR-100-3p**	**4.32**	**19.96**	**1.26E-05**	**miR-100-3p (miRNAs w/seed AAGCUUG)**
**13**	**hsa-miR-12135**	**4.31**	**19.82**	**6.95E-05**	**miR-12135 (miRNAs w/seed AAAGGUU)**

14	hsa-miR-371a-5p	4.29	19.51	9.49E-05	miR-293-5p (and other miRNAs w/seed CUCAAAC)
15	hsa-miR-518c-3p	4.25	18.99	1.00E-04	miR-518a-3p (and other miRNAs w/seed AAAGCGC)
16	hsa-miR-515-5p	4.05	16.57	7.08E-05	miR-515-5p (and other miRNAs w/seed UCUCCAA)
17	hsa-miR-31-3p	3.98	15.73	4.00E-04	miR-31-3p (and other miRNAs w/seed GCUAUGC)
18	hsa-miR-520f-3p	3.85	14.39	7.00E-04	miR-302c-3p (and other miRNAs w/seed AGUGCUU)
19	hsa-miR-516a-5p	3.66	12.67	1.00E-04	miR-516a-5p (miRNAs w/seed UCUCGAG)
20	hsa-miR-518f-3p	3.49	11.26	1.40E-03	miR-518a-3p (and other miRNAs w/seed AAAGCGC)
21	hsa-miR-6781-3p	3.48	11.18	1.90E-03	miR-2682-3p (and other miRNAs w/seed GCCUCUU)
22	hsa-miR-1298-5p	3.32	10.02	9.00E-04	miR-1298-5p (and other miRNAs w/seed UCAUUCG)
23	hsa-miR-31-5p	3.32	9.98	7.01E-09	miR-31-5p (and other miRNAs w/seed GGCAAGA)
24	hsa-miR-376b-3p	3.30	9.86	1.47E-05	miR-376a-3p (and other miRNAs w/seed UCAUAGA)
25	hsa-miR-106a-3p	3.26	9.58	4.00E-04	miR-106a-3p (and other miRNAs w/seed UGCAAUG)
26	hsa-miR-376c-5p	3.16	8.93	3.00E-03	miR-376b-5p (and other miRNAs w/seed GUGGAUA)
27	hsa-miR-517c-3p	3.07	8.41	4.70E-03	miR-517a-3p (and other miRNAs w/seed UCGUGCA)
28	hsa-miR-517a-3p	2.89	7.39	2.30E-03	miR-517a-3p (and other miRNAs w/seed UCGUGCA)
29	hsa-miR-518b	2.74	6.68	1.20E-03	miR-518a-3p (and other miRNAs w/seed AAAGCGC)
30	hsa-miR-18b-5p	2.67	6.38	9.00E-04	miR-18a-5p (and other miRNAs w/seed AAGGUGC)
31	hsa-miR-524-5p	2.57	5.95	1.93E-02	miR-520d-5p (and other miRNAs w/seed UACAAAG)
32	hsa-miR-4639-5p	2.545	5.84	2.03E-02	miR-4639-5p (miRNAs w/seed UGCUAAG)
33	hsa-miR-376b-5p	2.41	5.31	4.10E-03	miR-376b-5p (and other miRNAs w/seed GUGGAUA)
34	hsa-miR-105-5p	2.28	4.86	3.89E-02	miR-105-5p (and other miRNAs w/seed CAAAUGC)
35	hsa-miR-3195	2.27	4.81	6.40E-03	miR-3195 (miRNAs w/seed GCGCCGG)
36	hsa-miR-1323	2.27	4.81	2.76E-02	miR-548o-3p (and other miRNAs w/seed CAAAACU)
37	hsa-miR-376a-3p	2.28	4.71	2.83E-07	miR-376a-3p (and other miRNAs w/seed UCAUAGA)
38	hsa-miR-138-5p	2.22	4.66	2.47E-03	miR-138-5p (miRNAs w/seed GCUGGUG)
39	hsa-miR-143-5p	2.19	4.57	2.68E-05	miR-143-5p (and other miRNAs w/seed GUGCAGU)
40	hsa-miR-34a-5p	2.19	4.56	4.06E-07	miR-34a-5p (and other miRNAs w/seed GGCAGUG)
41	hsa-miR-299-5p	2.16	4.47	6.00E-04	miR-299a-5p (and other miRNAs w/seed GGUUUAC)
42	hsa-miR-539-3p	1.99	3.99	4.00E-03	miR-485-3p (and other miRNAs w/seed UCAUACA)
43	hsa-miR-222-5p	1.90	3.73	3.00E-02	miR-222-5p (miRNAs w/seed UCAGUAG)
44	hsa-miR-3168	1.86	3.63	1.00E-03	miR-3168 (miRNAs w/seed AGUUCUA)
45	hsa-miR-503-3p	1.85	3.60	3.00E-02	miR-503-3p (miRNAs w/seed GGGUAUU)
46	hsa-miR-154-5p	1.85	3.59	1.00E-03	miR-154-5p (miRNAs w/seed AGGUUAU)
47	hsa-miR-136-5p	1.84	3.59	1.00E-04	miR-136-5p (miRNAs w/seed CUCCAUU)
48	hsa-miR-218-5p	1.75	3.36	3.00E-04	miR-218-5p (and other miRNAs w/seed UGUGCUU)
49	hsa-miR-15a-3p	1.72	3.29	3.00E-02	miR-15a-3p (miRNAs w/seed AGGCCAU)
50	hsa-miR-3620-5p	1.70	3.26	3.00E-02	miR-1587 (and other miRNAs w/seed UGGGCUG)
51	hsa-miR-199b-5p	1.70	3.26	2.00E-04	miR-199a-5p (and other miRNAs w/seed CCAGUGU)
52	hsa-miR-363-5p	1.67	3.17	4.00E-02	miR-363-5p (and other miRNAs w/seed GGGUGGA)
53	hsa-miR-21-3p	1.56	2.95	2.00E-03	miR-122b-3p (and other miRNAs w/seed AACACCA)
54	hsa-miR-20b-5p	1.48	2.79	1.74E-05	miR-17-5p (and other miRNAs w/seed AAAGUGC)
55	hsa-miR-424-5p	1.46	2.76	3.73E-05	miR-16-5p (and other miRNAs w/seed AGCAGCA)
56	hsa-miR-125b-5p	1.48	2.73	2.53E-05	miR-125b-5p (and other miRNAs w/seed CCCUGAG)
57	hsa-miR-135a-5p	1.42	2.68	4.00E-02	miR-135a-5p (and other miRNAs w/seed AUGGCUU)
58	hsa-miR-494-3p	1.37	2.59	1.00E-03	miR-494-3p (miRNAs w/seed GAAACAU)
59	hsa-miR-379-3p	1.37	2.58	3.00E-02	miR-411-3p (and other miRNAs w/seed AUGUAAC)
60	hsa-miR-125b-1-3p	1.37	2.58	1.35E-02	miR-125b-1-3p (miRNAs w/seed CGGGUUA)
61	hsa-miR-199a-5p	1.32	2.50	3.06E-05	miR-199a-5p (and other miRNAs w/seed CCAGUGU)
62	hsa-miR-455-5p	1.32	2.49	2.00E-03	miR-455-5p (and other miRNAs w/seed AUGUGCC)
63	hsa-miR-411-5p	1.31	2.48	1.00E-03	miR-411-5p (and other miRNAs w/seed AGUAGAC)
64	hsa-miR-100-5p	1.27	2.41	2.00E-03	miR-100-5p (and other miRNAs w/seed ACCCGUA)
65	hsa-miR-542-3p	1.26	2.39	6.00E-03	miR-542-3p (miRNAs w/seed GUGACAG)

**66**	**hsa-miR-377-3p**	**1.23**	**2.34**	**8.00E-03**	**miR-377-3p (miRNAs w/seed UCACACA)**
**67**	**hsa-miR-18a-5p**	**1.15**	**2.22**	**2.00E-04**	**miR-18a-5p (and other miRNAs w/seed AAGGUGC)**
**68**	**hsa-miR-493-3p**	**1.14**	**2.21**	**3.00E-03**	**miR-493-3p (miRNAs w/seed GAAGGUC)**
**69**	**hsa-miR-1277-5p**	**1.10**	**2.15**	**1.90E-02**	**miR-1277-5p (miRNAs w/seed AAUAUAU)**
**70**	**hsa-miR-136-3p**	**1.04**	**2.05**	**2.40E-02**	**miR-136-3p (miRNAs w/seed AUCAUCG)**
**71**	**hsa-miR-149-5p**	**1.03**	**2.04**	**4.30E-02**	**miR-149-5p (miRNAs w/seed CUGGCUC)**
**72**	**hsa-miR-145-3p**	**1.02**	**2.02**	**4.20E-02**	**miR-145-3p (miRNAs w/seed GAUUCCU)**
**73**	**hsa-miR-145-5p**	**1.01**	**2.01**	**2.00E-03**	**miR-145-5p (and other miRNAs w/seed UCCAGUU)**
74	hsa-miR-6805-5p	-1.04	-2.06	1.60E-02	miR-3104-5p (and other miRNAs w/seed AGGGGGC)
75	hsa-miR-206	-1.18	-2.26	8.00E-03	miR-1-3p (and other miRNAs w/seed GGAAUGU)
76	hsa-miR-197-5p	-1.21	-2.31	1.80E-02	miR-197-5p (and other miRNAs w/seed GGGUAGA)
77	hsa-miR-1292-5p	-1.24	-2.37	4.90E-02	miR-1247-3p (and other miRNAs w/seed GGGAACG)
78	hsa-miR-548l	-1.26	-2.39	3.80E-02	miR-548l (miRNAs w/seed AAAGUAU)
79	hsa-miR-181c-5p	-1.31	-2.49	2.30E-02	miR-181a-5p (and other miRNAs w/seed ACAUUCA)
80	hsa-miR-412-5p	-1.40	-2.64	2.80E-02	miR-412-5p (miRNAs w/seed GGUCGAC)
81	hsa-miR-548b-5p	-1.42	-2.69	3.80E-02	miR-548h-5p (and other miRNAs w/seed AAAGUAA)
82	hsa-miR-3150b-3p	-1.47	-2.77	2.10E-02	miR-3150b-3p (and other miRNAs w/seed GAGGAGA)
83	hsa-miR-6891-5p	-1.50	-2.83	1.60E-02	miR-12118 (and other miRNAs w/seed AAGGAGG)
84	hsa-miR-151b	-1.53	-2.93	3.20E-02	miR-151-5p (and other miRNAs w/seed CGAGGAG)
85	hsa-miR-216a-5p	-1.55	-2.93	2.90E-02	miR-216a-5p (miRNAs w/seed AAUCUCA)
86	hsa-miR-202-5p	-1.59	-3.02	2.50E-02	miR-202-5p (miRNAs w/seed UCCUAUG)
87	hsa-miR-548o-3p	-1.65	-3.16	1.60E-02	miR-548o-3p (and other miRNAs w/seed CAAAACU)
88	hsa-miR-378d	-1.68	-3.21	3.00E-02	miR-378a-3p (and other miRNAs w/seed CUGGACU)
89	hsa-miR-6785-5p	-1.77	-3.42	3.80E-02	miR-149-3p (and other miRNAs w/seed GGGAGGG)
90	hsa-miR-4648	-1.79	-3.47	4.40E-02	miR-4648 (miRNAs w/seed GUGGGAC)
91	hsa-miR-450a-2-3p	-1.91	-3.76	1.20E-02	miR-450b-3p (and other miRNAs w/seed UUGGGGA)
92	hsa-miR-4745-5p	-1.95	-3.85	4.90E-02	miR-4481 (and other miRNAs w/seed GAGUGGG)
93	hsa-miR-184	-2.11	-4.32	6.00E-03	miR-184 (and other miRNAs w/seed GGACGGA)
94	hsa-miR-219a-5p	-2.12	-4.36	1.80E-02	miR-219a-5p (and other miRNAs w/seed GAUUGUC)
95	hsa-miR-128-1-5p	-2.13	-4.39	1.90E-02	miR-128-1-5p (and other miRNAs w/seed GGGGCCG)
96	hsa-miR-6516-5p	-2.22	-4.67	7.00E-03	miR-6516-5p (miRNAs w/seed UUGCAGU)
97	hsa-miR-5189-5p	-2.52	-5.74	1.00E-03	miR-1285-3p (and other miRNAs w/seed CUGGGCA)
98	hsa-miR-378g	-2.53	-5.79	3.00E-03	miR-378g (miRNAs w/seed CUGGGCU)
99	hsa-let-7f-2-3p	-2.79	-6.94	2.00E-04	let-7f-2-3p (and other miRNAs w/seed UAUACAG)
100	hsa-miR-6509-5p	-3.05	-8.33	8.00E-03	miR-6509-5p (miRNAs w/seed UUAGGUA)

Supplementary Table S4: Other ncRNAs differential expression analysis: severe symptoms *vs.* mild symptoms cases

N#	Name	Chromosome	Region	Max group mean	Log_2 fold change	Fold change	P-value
**1**	**MT-TG**	**MT**	**9991..10058**	**11827**	**3.57**	**11.86**	**1.74E-06**
**2**	**MT-TD**	**MT**	**7518..7585**	**19823**	**1.82**	**3.53**	**2.80E-03**
**3**	**MT-TF**	**MT**	**577..647**	**4269**	**1.81**	**3.52**	**8.76E-03**
**4**	**MT-TW**	**MT**	**5512..5579**	**682**	**1.79**	**3.46**	**1.04E-02**
**5**	**MT-TR**	**MT**	**10405..10469**	**3039**	**1.20**	**2.30**	**4.15E-02**
6	MT-TN	MT	c(5657..5729)	3054	-1.55	-2.92	1.93E-02
7	MT-RNR1	MT	648..1601	2516	-1.79	-3.46	6.20E-04
8	MT-TM	MT	4402..4469	92425	-2.69	-6.44	8.47E-05
**9**	**AL031595.3**	**22**	**44139365..44153626**	**56**	**3.74**	**13.32**	**1.44E-07**

**10**	**LINC02739**	**11**	**c(59560298..59566283)**	**2466**	**2.88**	**7.34**	**2.23E-07**
**11**	**AL928646.1**	**X**	**109054542..109054590**	**1001**	**2.10**	**4.30**	**2.50E-03**
**12**	**AP003499.4**	**11**	**93721542..93721621**	**145**	**1.76**	**3.38**	**4.38E-02**
**13**	**AC051619.7**	**15**	**c(45200325..45200632)**	**2214**	**1.13**	**2.19**	**2.14E-02**
**14**	**AC108134.2**	**16**	**c(3156736..3157483)**	**83**	**1.06**	**2.09**	**3.80E-02**
15	AP005900.1	18	7995570..7995785	70	-1.14	-2.21	4.22E-02
16	AL135938.1	20	c(16670641..16670742)	14834	-1.21	-2.32	4.62E-02
17	AL391647.2	X	41316488..41316589	1714	-1.22	-2.34	3.91E-02
18	AC011603.1	12	c(49132853..49132947)	104	-1.29	-2.45	4.66E-02
19	AC012377.1	15	c(39922782..39922886)	103	-1.30	-2.46	3.29E-02
20	AL359759.1	10	c(100398352..100398446)	309	-1.42	-2.68	4.92E-02
21	AC091057.2	15	30673750..30673843	192	-1.49	-2.82	3.71E-02
22	AC132825.2	17	c(22524563..22525330)	194	-1.61	-3.06	2.90E-02
23	FP671120.8	21	c(8254592..8255514)	549	-1.88	-3.67	4.34E-02
24	AC090527.1	15	45591984..45592085	74	-2.05	-4.14	4.96E-03
25	AL035087.1	6	c(99642237..99642347)	60	-2.32	-5.00	1.12E-02
26	FP671120.4	21	8256781..8256933	5756	-2.51	-5.71	8.95E-06
27	AL109920.1	6	106454828..106454929	936	-4.98	-31.64	3.34E-07
28	LINC01921	2	216859948..216871643	69	-6.71	-104.58	2.06E-07

c: complement

Supplementary Table S5: Ingenuity Pathways Analysis (IPA) ASD *vs.* Control.

ID	Molecules in Network	Score	Focus Molecules	Top Diseases and Functions
1	ACSL6, **AGO2**, CNOT6, GFI1, Gnasas1, miR-101-3p, miR-122-5p, miR-125b-2-3p, mir-126, mir-130, mir-134, mir-188, miR-192-5p, miR-196a-5p, miR-199a-5p, miR-19b-3p, miR-221-3p, miR-28-3p, miR-291a-3p, mir-302, miR-3118, miR-328-3p, mir-335, miR-335-5p, miR-4446-3p, miR-660-5p, miR-96-5p, MKRN1, **NR4A2**, TENT2	35	16	[Cancer, Organismal Injury and Abnormalities, Reproductive System Disease]
2	ADAMTS14, BLACAT1, **BRAF**, CDKN2A, Gnasas1, let-7a-5p, miR-100-5p, miR-1255b-5p, miR-127-3p, miR-141-3p, miR-144-3p, miR-144-5p, miR-150-3p, miR-17-5p, miR-185-5p, miR-194-5p, mir-290, miR-29b-3p, mir-302, miR-375-3p, miR-485-5p, mir-515, miR-744-5p, miR-7a-5p, MYC, PAX3-FOXO1, Smad2/3, SMAD6/7, Tgf beta, TUSC2	35	16	[Cancer, Organismal Injury and Abnormalities, Reproductive System Disease]
3	calcifediol, CD24, DDX60L, **DKK1**, **EPHB6**, HERC6, KIF23, miR-100-5p, miR-10396b-5p, miR-1180-3p, miR-1285-3p, miR-148b-5p, miR-16-5p, miR-182-5p, miR-183-5p, mir-1908, miR-193b-5p, miR-23a-5p, mir-335, miR-485-3p, mir-497, miR-542-3p, miR-584-5p, **NTRK3**, RECK, RPS15, RTP4, SLFN5, **TP53**, tretinoin, TRMT13	27	13	[Neurological Disease, Organismal Injury and Abnormalities, Psychological Disorders]

Supplementary Table S6: Ingenuity Pathways Analysis (IPA) networks ASD with severe symptoms *vs.* mild.

ID	Molecules in Network	Score	Focus Molecules	Top Diseases and Functions
1	**BRAF**, ERBB2, let-7f-2-3p, miR-106a-3p, mir-1231, miR-1285-3p, mir-138, miR-138-5p, miR-149-5p, miR-151-5p, mir-154, miR-154-5p, mir-17, mir-184, mir-187, miR-187-3p, miR-18a-5p, mir-197, miR-197-5p, miR-216a-5p, mir-25, miR-291a-3p, mir-302, miR-302a-5p, miR-302b-5p, miR-302c-5p, miR-377-3p, miR-485-3p, miR-494-3p, OIP5-AS1, **PTEN**, RNF149, SRC (family), UCA1	40	19	[Cancer, Organismal Injury and Abnormalities, Reproductive System Disease]
2	calcifediol, **EGFR**, HARS1, **IGF1R**, mir-10, miR-100-3p, miR-100-5p, miR-105-5p, miR-125b-1-3p, mir-136, miR-136-3p, miR-136-5p, miR-16-5p, mir-25, mir-299, miR-299a-5p, mir-31, miR-31-3p, miR-31-5p, miR-3104-5p, mir-322, mir-368, miR-378a-3p, mir-379, miR-411-3p, miR-411-5p, mir-542, miR-542-3p, miR-92a-3p, PAX3-FOXO1, PIK3C2B, PTPN7, RECK, RTL1, **TLR2**	32	16	[Cancer, Gastrointestinal Disease, Organismal Injury and Abnormalities]

3	AGO1, **AGO2**, ALOX5AP, **ARGONAUTE**, DDX20, FGF16, **MEF2A**, miR-100-5p, miR-122b-3p, miR-135a-5p, miR-143-5p, mir-154, miR-15a-3p, miR-199a-5p, miR-21-5p, miR-219a-5p, miR-3150b-3p, mir-363, miR-363-5p, miR-455-5p, miR-493-3p, miR-494-3p, miR-515-5p, miR-548o-3p, miR-92a-3p, ZBP1	29	15	[Gene Expression, Organismal Injury and Abnormalities, Reproductive System Disease]
